# Supplementary material for: Chrysomycin A Regulates Proliferation and Apoptosis of Neuroglioma Cells via the Akt/GSK-3β Signaling Pathway In Vivo and In Vitro
Source: Mar Drugs. 2023 May 27;21(6):329. doi: 10.3390/md21060329 (PMC10304729; doi:10.3390/md21060329)
Supplement: Supplementary file 1 [file marinedrugs-21-00329-s001.zip › marinedrugs-2377544-supplementary.pdf]

Supplementary information Table S1: DEGs regulated by Chr-A were obtained from RNA-seq analysis of tumor tissues

Chr A-Related Target	fold changes	p-values
CTSD	46.1068	0.0019
ITGB1	28.9217	0.0000
RPS5	22.2056	0.0252
KPNB1	19.9508	0.0002
IMMT	13.0924	0.0303
AIMP1	12.6145	0.0001
SLC43A3	10.7698	0.0209
MLH1	10.0699	0.0004
PRPSAP1	8.8754	0.0008
IP6K1	8.1634	0.0000
AVL9	8.1329	0.0032
ARHGAP22	8.0901	0.0002
AES	8.0083	0.0000
PITPNB	7.6509	0.0324
TP53	7.2254	0.0004
GNPDA1	7.2235	0.0050
TNFAIP2	7.1712	0.0088
TOR1AIP1	6.9239	0.0039
SSBP3	6.8031	0.0015
INTS14	6.7593	0.0010
UBAC2	6.7454	0.0049
ACACA	6.1180	0.0000
ZYX	6.0630	0.0224
DIDO1	5.9091	0.0000
METTL7A	5.8520	0.0261
PMP22	5.8439	0.0010
DICER1	5.6950	0.0005
RNF41	5.4313	0.0012
CRLS1	5.3438	0.0001
VPS26A	5.2147	0.0174
ELP3	4.8439	0.0086
RPS15	4.8338	0.0307
CSNK1G3	4.7851	0.0002
CADPS	4.7562	0.0431
ANKRD28	4.7410	0.0201
DCAF5	4.6952	0.0002
GOLGA2	4.6052	0.0002

PARP1	4.5876	0.0006
SERP1	4.4603	0.0307
TMEM9	4.4258	0.0018
SAMD11	4.3750	0.0089
FRMD6	4.2764	0.0424
STK16	4.2471	0.0197
ADGRL2	4.2462	0.0033
PML	4.1717	0.0037
FZD6	4.1302	0.0023
BGLAP	4.0841	0.0123
TEN1	4.0764	0.0404
CDC25C	4.0565	0.0337
ZMYND8	3.9436	0.0404
DMAP1	3.9036	0.0073
RPL35A	3.8483	0.0452
ZBTB14	3.8380	0.0097
PCID2	3.8369	0.0412
TXNDC9	3.8243	0.0238
C12orf75	3.7689	0.0461
MRPL34	3.7539	0.0433
PELP1	3.7111	0.0109
COMMD7	3.6617	0.0142
CCDC14	3.5944	0.0021
CCT7	3.5550	0.0169
CCDC113	3.5416	0.0373
NIF3L1	3.5085	0.0204
TP53BP1	3.4916	0.0459
PON2	3.4493	0.0159
RIDA	3.4453	0.0440
PHC2	3.4316	0.0160
PRKCD	3.4097	0.0014
ZNF688	3.4011	0.0058
DCAKD	3.3876	0.0405
CCL24	3.3855	0.0392
CDC45	3.3531	0.0085
SHKBP1	3.3273	0.0376
ELOVL5	3.3134	0.0182
ANAPC11	3.2797	0.0145
RAB4B	3.2603	0.0114
CYFIP1	3.2185	0.0144
YY1AP1	3.2152	0.0205
ZC3HC1	3.1879	0.0133
GTF3C5	3.1688	0.0009

ZNF236	3.1671	0.0002
CBWD1	3.1414	0.0094
RND3	3.1138	0.0089
WDR61	3.0982	0.0340
FRYL	3.0953	0.0100
RNPEPL1	3.0913	0.0417
RAD51B	3.0631	0.0039
COL4A6	3.0367	0.0199
SGMS1	3.0361	0.0000
LANCL1	3.0349	0.0133
PXK	3.0212	0.0113
MGA	3.0090	0.0001
PLOD2	2.9942	0.0186
CLSPN	2.9478	0.0285
MED27	2.9313	0.0293
ATP11C	2.9175	0.0233
AC099811.2	2.8977	0.0095
ADGRE1	2.8977	0.0298
FLNC	2.8970	0.0400
TSEN15	2.8897	0.0199
AURKA	2.8857	0.0294
AGBL5	2.8767	0.0351
WDR74	2.8686	0.0454
DPM3	2.8525	0.0060
ZCCHC10	2.8458	0.0219
RNF8	2.8346	0.0006
EXOSC1	2.8339	0.0068
USP47	2.8227	0.0173
TLK1	2.8102	0.0030
C5orf24	2.8067	0.0308
BCL2L1	2.7959	0.0299
FERMT3	2.7785	0.0004
TAF9	2.7771	0.0007
HNRNPDL	2.7186	0.0182
APOBEC3B	2.7008	0.0011
MIF4GD	2.6855	0.0000
MRPL23	2.6667	0.0102
CDH18	2.6622	0.0000
GPR107	2.6479	0.0093
MXI1	2.6302	0.0123
BCKDK	2.6185	0.0279
BTBD10	2.6081	0.0446
ZSWIM7	2.5997	0.0079

CCNC	2.5989	0.0022
YPEL3	2.5953	0.0242
SYTL3	2.5858	0.0064
BDKRB1	2.5755	0.0119
TNFRSF19	2.5725	0.0184
GPATCH4	2.5418	0.0441
C11orf80	2.5303	0.0000
HIST1H3B	2.5279	0.0059
MTMR14	2.5270	0.0297
KCNMA1	2.5253	0.0011
SCML2	2.5173	0.0019
TROAP	2.5091	0.0050
ZNF749	2.5068	0.0026
LSM4	2.5065	0.0344
SLC4A2	2.5057	0.0446
HYI	2.4969	0.0011
N4BP2	2.4903	0.0296
BTBD11	2.4876	0.0312
ELP5	2.4671	0.0146
RPUSD3	2.4476	0.0498
ATP6V0E2	2.4454	0.0379
FDFT1	2.4299	0.0184
NUSAP1	2.4267	0.0295
MTMR12	2.4221	0.0083
RPS3A	2.4035	0.0438
FBXO8	2.3976	0.0333
EWSR1	2.3975	0.0337
PTK2	2.3971	0.0076
HIST1H1B	2.3907	0.0020
NSMCE2	2.3801	0.0350
HIST1H1E	2.3788	0.0118
SCG5	2.3755	0.0390
CCNA1	2.3668	0.0001
HIST2H2AC	2.3418	0.0134
DCLRE1C	2.3253	0.0173
NELFCD	2.3064	0.0001
MFGE8	2.3055	0.0067
DOCK10	2.2993	0.0426
KLC4	2.2961	0.0056
HAUS4	2.2901	0.0007
GOLGA7	2.2826	0.0230
CSNK2B	2.2789	0.0071
SHROOM2	2.2784	0.0479

CD58	2.2750	0.0072
RBMX	2.2738	0.0157
COQ10B	2.2692	0.0018
FANCI	2.2647	0.0011
C14orf2	2.2629	0.0274
ERCC8	2.2432	0.0103
RSBN1	2.2350	0.0036
RPP40	2.2312	0.0401
RPL6	2.2306	0.0173
SLC38A9	2.2236	0.0038
RERE	2.2190	0.0133
PERP	2.2179	0.0060
PREX1	2.2150	0.0057
PER3	2.2116	0.0019
SOX13	2.2109	0.0026
KCTD6	2.2048	0.0313
MYO6	2.2000	0.0013
TMEM267	2.1962	0.0037
ZNF586	2.1958	0.0155
STX10	2.1908	0.0185
SLITRK6	2.1888	0.0078
HADH	2.1866	0.0106
TMEM100	2.1859	0.0071
C11orf74	2.1849	0.0250
RBMS3	2.1816	0.0038
KLRC2	2.1788	0.0043
EHMT2	2.1787	0.0222
LPP	2.1780	0.0087
GNPAT	2.1755	0.0003
EXOSC2	2.1644	0.0081
CACTIN	2.1570	0.0299
PTTG1	2.1561	0.0148
LY6E	2.1507	0.0074
DEPDC1	2.1417	0.0499
ZFP90	2.1374	0.0406
CRTC2	2.1370	0.0137
AKR1C2	2.1324	0.0087
CAST	2.1320	0.0464
HTR7	2.1296	0.0214
RBCK1	2.1287	0.0009
TBL3	2.1265	0.0431
NLN	2.1178	0.0378
EEF1B2	2.1068	0.0057

CLYBL	2.1024	0.0040
CALD1	2.0982	0.0187
PDE4D	2.0819	0.0412
AKAP8L	2.0764	0.0487
SDF4	2.0688	0.0301
RANGRF	2.0687	0.0086
FDPS	2.0639	0.0199
PBX3	2.0592	0.0485
C1orf112	2.0529	0.0086
CINP	2.0526	0.0475
OGDH	2.0526	0.0408
DENND4C	2.0482	0.0162
HMCN2	2.0448	0.0383
MAIP1	2.0417	0.0395
ZFYVE19	2.0366	0.0074
SLC39A13	2.0263	0.0487
INVS	2.0193	0.0328
AEBP2	2.0178	0.0138
AADAT	2.0176	0.0016
SMG7	2.0175	0.0224
PHF19	2.0166	0.0139
ZDHHC8	2.0164	0.0122
RNF146	2.0136	0.0024
SNURF	2.0136	0.0279
WARS	2.0129	0.0169
MRE11	2.0126	0.0074
ZNF138	2.0017	0.0063
ANKRD35	2.0016	0.0076
FOXP2	1.9986	0.0376
PRPSAP2	1.9979	0.0488
MTHFD2L	1.9955	0.0394
HMBS	1.9916	0.0391
PHF6	1.9878	0.0327
PPP1R7	1.9874	0.0015
PTBP1	1.9870	0.0474
IFI27L1	1.9794	0.0326
RACK1	1.9782	0.0016
SLC6A15	1.9778	0.0127
ZNHIT3	1.9759	0.0055
LIN37	1.9747	0.0309
FOXC2	1.9743	0.0493
SHROOM1	1.9721	0.0157
FBXO28	1.9715	0.0150

CXXC1	1.9702	0.0176
ATP5G1	1.9626	0.0239
SNRNP35	1.9590	0.0007
PRPS2	1.9589	0.0007
GMNN	1.9588	0.0112
CDCA7L	1.9588	0.0257
ZFP1	1.9575	0.0022
NDUFV1	1.9572	0.0160
KNTC1	1.9569	0.0043
PHF23	1.9566	0.0197
ZMYM6	1.9550	0.0075
CUTA	1.9534	0.0248
ATP13A2	1.9502	0.0375
FANCB	1.9493	0.0222
HBS1L	1.9436	0.0041
RPL17	1.9425	0.0357
TTC13	1.9421	0.0185
IDH1	1.9417	0.0342
ADSL	1.9340	0.0091
DDX39A	1.9339	0.0459
MELK	1.9337	0.0420
LYPLA2	1.9308	0.0375
CREB3L2	1.9224	0.0498
CCNB1	1.9208	0.0295
OGFR	1.9185	0.0241
CSDE1	1.9070	0.0077
HOXA3	1.9022	0.0028
AKAP8	1.9002	0.0016
IRF3	1.8988	0.0132
MED17	1.8971	0.0262
RNASEH2B	1.8945	0.0484
PPA2	1.8913	0.0383
COQ8B	1.8900	0.0186
SSBP1	1.8885	0.0077
DCXR	1.8837	0.0093
BAIAP2	1.8798	0.0161
OSBPL10	1.8780	0.0103
MBD6	1.8767	0.0302
MCTP1	1.8751	0.0226
CCDC124	1.8698	0.0439
BROX	1.8648	0.0460
NELFE	1.8648	0.0011
TMEM106C	1.8648	0.0418

DUSP22	1.8642	0.0049
SLC39A14	1.8621	0.0314
USP21	1.8590	0.0380
MND1	1.8523	0.0331
PRKAR2B	1.8521	0.0444
KYAT1	1.8497	0.0397
NELFA	1.8487	0.0276
ANAPC7	1.8444	0.0004
ARMC10	1.8430	0.0048
RPL26	1.8402	0.0234
RELB	1.8381	0.0232
TRPT1	1.8367	0.0219
HIST1H3E	1.8304	0.0015
ACAT2	1.8295	0.0279
C3AR1	1.8273	0.0149
TM4SF1	1.8254	0.0155
ZNF644	1.8248	0.0095
RNPS1	1.8241	0.0407
DNER	1.8225	0.0022
NPC1	1.8202	0.0337
KIF2A	1.8181	0.0250
ANKRD16	1.8151	0.0044
FAM46A	1.8151	0.0246
PMPCB	1.8134	0.0007
RHBDD2	1.8089	0.0385
ZNF286A	1.8075	0.0032
CSF3	1.8060	0.0262
GNB1L	1.8043	0.0121
ZNF440	1.8037	0.0382
CPT1C	1.7967	0.0166
ANKRD7	1.7951	0.0146
CCDC74A	1.7932	0.0395
DCUN1D2	1.7931	0.0022
APOPT1	1.7927	0.0227
PLA2G4A	1.7915	0.0399
ZMYND11	1.7882	0.0157
MRPS24	1.7875	0.0367
C6orf89	1.7860	0.0410
ULBP3	1.7850	0.0266
ITPK1	1.7848	0.0176
NAGA	1.7812	0.0397
INSIG1	1.7802	0.0222
ATG5	1.7796	0.0143

YIPF3	1.7795	0.0214
ITGB1BP1	1.7789	0.0391
PSMB8	1.7788	0.0313
SMN2	1.7782	0.0394
LCORL	1.7772	0.0445
DDHD2	1.7745	0.0426
H3F3A	1.7723	0.0214
GRAMD4	1.7717	0.0189
ILVBL	1.7707	0.0488
UFSP2	1.7706	0.0011
NAMPT	1.7684	0.0286
DYNC1LI2	1.7661	0.0000
RMDN1	1.7643	0.0174
NUMB	1.7639	0.0248
RPL36	1.7636	0.0119
SLC20A2	1.7619	0.0478
VEZF1	1.7612	0.0206
SUPT5H	1.7576	0.0385
RPRD1A	1.7562	0.0058
DNAAF3	1.7549	0.0375
SPOCK3	1.7540	0.0344
KCNMB3	1.7496	0.0199
EZH2	1.7477	0.0012
FAM60A	1.7451	0.0020
FBL	1.7418	0.0009
TAF1A	1.7408	0.0154
UBE2D3	1.7404	0.0343
TIMM23B	1.7386	0.0022
RNASE4	1.7382	0.0283
STXBP6	1.7372	0.0065
TRIML2	1.7365	0.0029
ZNF404	1.7354	0.0006
MPV17	1.7340	0.0069
BACH1	1.7334	0.0293
CLN3	1.7318	0.0148
NAB2	1.7316	0.0339
ZBTB44	1.7313	0.0094
TECR	1.7308	0.0336
SLC12A2	1.7303	0.0008
SSBP4	1.7270	0.0179
SMURF2	1.7259	0.0482
CLN8	1.7222	0.0470
CCNH	1.7214	0.0186

LRBA	1.7211	0.0037
KIF2C	1.7208	0.0044
PRR16	1.7184	0.0169
TOPORS	1.7170	0.0192
NEK2	1.7143	0.0079
EPB41	1.7129	0.0316
KLHL7	1.7124	0.0427
RBM4	1.7120	0.0399
MRPS18C	1.7112	0.0278
GALNT11	1.7070	0.0158
IDH3B	1.7062	0.0420
HNRNPH1	1.7043	0.0056
RPL7A	1.7024	0.0168
MAML3	1.7023	0.0275
HIST1H2BE	1.6994	0.0200
NSD2	1.6987	0.0317
RIN1	1.6984	0.0057
SP2	1.6976	0.0472
NLE1	1.6946	0.0170
TTK	1.6939	0.0222
BUB1	1.6931	0.0029
HNRNPC	1.6912	0.0342
CKS2	1.6896	0.0107
METTL22	1.6890	0.0111
CARD8	1.6855	0.0078
NEK11	1.6848	0.0281
ING5	1.6841	0.0083
TMEM205	1.6836	0.0041
CKS1B	1.6821	0.0345
KLC1	1.6796	0.0304
PCDHGB2	1.6782	0.0258
PIH1D1	1.6755	0.0381
SMIM30	1.6754	0.0001
ZNF35	1.6749	0.0063
PEX19	1.6731	0.0259
AGFG1	1.6729	0.0257
ANKRD49	1.6710	0.0119
PRUNE1	1.6700	0.0012
SLCO4A1	1.6698	0.0354
SERPINB2	1.6666	0.0135
SLC12A9	1.6643	0.0001
C19orf54	1.6633	0.0236
TMEM101	1.6632	0.0453

NFU1	1.6628	0.0340
SH3D21	1.6614	0.0037
RMND5B	1.6610	0.0392
C5orf34	1.6608	0.0065
RNF4	1.6603	0.0219
TMEM71	1.6586	0.0013
DHRS3	1.6580	0.0463
PCBP4	1.6576	0.0341
TMEM243	1.6535	0.0340
SCYL1	1.6526	0.0483
HIST1H3C	1.6525	0.0262
ZNF185	1.6517	0.0188
RPLP2	1.6512	0.0217
ARL10	1.6502	0.0186
DTX3	1.6488	0.0472
CENPA	1.6464	0.0110
IL17RC	1.6443	0.0091
FOS	1.6424	0.0367
ANXA1	1.6396	0.0317
RAD51C	1.6389	0.0048
NFE2L1	1.6368	0.0484
PAK4	1.6353	0.0465
MRPL54	1.6352	0.0072
ANAPC10	1.6296	0.0209
FAM83D	1.6280	0.0201
LYPLA1	1.6257	0.0296
SF3B2	1.6251	0.0421
ISG20	1.6244	0.0390
LSM1	1.6230	0.0296
SAAL1	1.6213	0.0150
TRIM47	1.6206	0.0187
SIGLEC15	1.6206	0.0058
GLT8D1	1.6194	0.0318
PFKFB4	1.6185	0.0374
HMOX2	1.6179	0.0012
MAPK9	1.6177	0.0189
ECSIT	1.6172	0.0358
PPP4R4	1.6169	0.0069
MRGBP	1.6163	0.0096
MRPL22	1.6161	0.0305
OSCP1	1.6156	0.0371
MPP6	1.6152	0.0023
ALDH7A1	1.6133	0.0333

UBE2C	1.6130	0.0080
PPFIA2	1.6126	0.0367
FHOD3	1.6119	0.0207
KTN1	1.6109	0.0257
SETDB1	1.6104	0.0471
YBEY	1.6104	0.0284
LDHA	1.6096	0.0021
IER5L	1.6092	0.0363
PMCH	1.6090	0.0170
MIEF1	1.6087	0.0276
EXOC1	1.6086	0.0469
ERG28	1.6084	0.0194
FOXL1	1.6077	0.0492
PPP2R3A	1.6067	0.0396
RPS8	1.6065	0.0010
NIPAL3	1.6057	0.0312
NDUFA5	1.6057	0.0176
ANG	1.6053	0.0329
HIST1H4J	1.6031	0.0031
CHMP2A	1.6016	0.0221
NOL10	1.5982	0.0032
RALBP1	1.5965	0.0369
CCDC148	1.5937	0.0166
SYNE3	1.5926	0.0157
MAD2L1BP	1.5915	0.0041
PRELID2	1.5913	0.0198
CAPS2	1.5908	0.0309
GTF3C2	1.5904	0.0289
C3orf14	1.5901	0.0282
ING1	1.5875	0.0249
PLCD4	1.5866	0.0284
RGS10	1.5845	0.0415
LETM2	1.5842	0.0242
RPS27A	1.5828	0.0447
NDUFA10	1.5806	0.0243
LRRC57	1.5794	0.0051
SPRY2	1.5791	0.0038
H2AFZ	1.5765	0.0194
WASF1	1.5761	0.0330
RGS20	1.5743	0.0105
WTAP	1.5737	0.0093
KIAA0586	1.5733	0.0251
GPX4	1.5726	0.0052

JOSD2	1.5711	0.0169
TSEN34	1.5708	0.0386
ADAM22	1.5680	0.0077
LIPE	1.5671	0.0099
PLSCR4	1.5661	0.0134
ARL6IP1	1.5657	0.0130
UCHL5	1.5649	0.0336
ERC1	1.5639	0.0367
DUSP23	1.5619	0.0391
MARK1	1.5618	0.0464
KBTBD2	1.5601	0.0231
C15ORF31	1.5599	0.0287
EMP3	1.5587	0.0072
RPUSD4	1.5586	0.0257
CPNE1	1.5579	0.0447
SERPINE2	1.5577	0.0275
MKRN2	1.5569	0.0319
WWOX	1.5564	0.0380
FAM189B	1.5562	0.0000
ATAD2	1.5559	0.0483
STEAP3	1.5556	0.0064
PRKCE	1.5553	0.0188
AVPI1	1.5548	0.0079
SIRT4	1.5526	0.0031
SH3BGR	1.5516	0.0342
CEPT1	1.5511	0.0345
DNM1L	1.5504	0.0235
MARCH9	1.5503	0.0106
DYNLL1	1.5498	0.0174
MRPL39	1.5491	0.0192
CHST11	1.5487	0.0377
MAPKAP1	1.5476	0.0461
ASAH2	1.5475	0.0000
ST3GAL3	1.5459	0.0147
SPCS2	1.5458	0.0006
TEX30	1.5440	0.0306
COMMD10	1.5440	0.0006
KLHDC2	1.5437	0.0300
NSMCE4A	1.5429	0.0257
MICAL3	1.5427	0.0147
ENOSF1	1.5414	0.0011
LRRC8C	1.5408	0.0464
PCNX1	1.5397	0.0411

YEATS4	1.5393	0.0227
CRACR2B	1.5390	0.0343
REPS1	1.5385	0.0392
NDEL1	1.5380	0.0003
TMA16	1.5380	0.0283
SAP30	1.5372	0.0259
YWHAE	1.5370	0.0109
PLEC	1.5364	0.0177
ZNF287	1.5364	0.0499
MORN4	1.5363	0.0206
RBM4B	1.5361	0.0258
KARS	1.5360	0.0029
PTPN12	1.5352	0.0442
LTA4H	1.5339	0.0307
LDAH	1.5330	0.0302
SYNRG	1.5328	0.0249
FAM72B	1.5297	0.0205
RNASEH2C	1.5295	0.0173
ARHGAP11A	1.5285	0.0025
COQ3	1.5283	0.0378
UBTF	1.5279	0.0498
YBX1	1.5273	0.0479
FAM133B	1.5244	0.0297
PRPF31	1.5244	0.0432
TRIOBP	1.5239	0.0468
CSTF3	1.5209	0.0179
NGDN	1.5195	0.0443
BICRA	1.5189	0.0074
XPO1	1.5187	0.0156
SREK1IP1	1.5184	0.0039
HELLS	1.5168	0.0437
SEC63	1.5152	0.0030
ZNF655	1.5151	0.0023
MSANTD2	1.5147	0.0207
GCFC2	1.5131	0.0312
THAP10	1.5123	0.0253
ELAVL2	1.5105	0.0054
ZNF684	1.5102	0.0147
TKFC	1.5100	0.0089
CPSF1	1.5094	0.0009
STAMBP	1.5086	0.0279
PAPOLA	1.5076	0.0255
TIMP1	1.5067	0.0100

HERC4	1.5064	0.0161
SMPDL3A	1.5060	0.0322
ZMYND10	1.5059	0.0380
USP3	1.5057	0.0491
PLGRKT	1.5052	0.0130
ZSCAN20	1.5050	0.0022
PRR7	1.5040	0.0028
ELK4	1.5033	0.0149
ZNF587	1.5024	0.0416
SRD5A3	1.5013	0.0033
DEPDC1B	1.5006	0.0166
NDC80	1.5003	0.0217
CNN3	0.0556	0.0099
C6orf48	0.0793	0.0000
BGN	0.0910	0.0271
EDIL3	0.1084	0.0171
PTK7	0.1245	0.0002
RAB23	0.1622	0.0079
OS9	0.1652	0.0309
STOML1	0.1695	0.0319
SLN	0.1699	0.0458
MCM3	0.1827	0.0260
ACTG2	0.1923	0.0323
SFRP4	0.1948	0.0245
BCL2L13	0.1993	0.0032
PRPS1	0.2040	0.0002
FAM32A	0.2052	0.0001
ACTA2	0.2086	0.0069
RNF182	0.2098	0.0079
MYL9	0.2122	0.0240
CCAR1	0.2236	0.0007
CDK4	0.2297	0.0467
ZNF532	0.2316	0.0052
DDX47	0.2352	0.0206
STK40	0.2360	0.0345
SEPHS1	0.2386	0.0003
SUPT6H	0.2412	0.0286
NUP153	0.2414	0.0484
ABHD12	0.2460	0.0225
ATG16L1	0.2474	0.0207
FHL1	0.2479	0.0199
TPM1	0.2535	0.0176
CENPT	0.2650	0.0009

PLSCR3	0.2657	0.0343
LINC00094	0.2673	0.0003
CNN2	0.2713	0.0027
COL1A1	0.2788	0.0184
SLC12A4	0.2803	0.0472
MAGED1	0.2808	0.0169
PGRMC2	0.2878	0.0192
LRRC15	0.2880	0.0448
PARP10	0.2948	0.0045
BSCL2	0.2949	0.0158
ANAPC15	0.2949	0.0229
MME	0.2969	0.0325
CLIC3	0.2974	0.0111
ERP29	0.2989	0.0036
KDM6A	0.3017	0.0004
FIBIN	0.3024	0.0304
SEC31A	0.3087	0.0156
GPNMB	0.3124	0.0262
SELENOM	0.3171	0.0379
ITGA11	0.3189	0.0047
KCNS3	0.3212	0.0418
PLXDC1	0.3233	0.0493
SNRNP25	0.3272	0.0014
C1QTNF1	0.3273	0.0175
STRA6	0.3280	0.0051
TNC	0.3285	0.0239
PPP4R1	0.3293	0.0343
TMSB15B	0.3315	0.0094
SLC38A6	0.3325	0.0044
TFIP11	0.3345	0.0148
THBS3	0.3372	0.0176
ZFAND2A	0.3386	0.0250
CHRNA1	0.3411	0.0146
SPI1	0.3437	0.0334
CD164	0.3439	0.0005
LMOD1	0.3506	0.0051
MINK1	0.3510	0.0364
HSPG2	0.3524	0.0227
PMEPA1	0.3531	0.0098
NFATC3	0.3539	0.0004
DNASE1L1	0.3541	0.0002
UCP2	0.3547	0.0206
URGCP	0.3559	0.0471

HSPB3	0.3564	0.0084
RAB15	0.3575	0.0121
RHOC	0.3580	0.0011
DLG1	0.3589	0.0059
OSBPL9	0.3590	0.0342
DDX11	0.3592	0.0337
RNF38	0.3605	0.0318
ZNF17	0.3667	0.0023
ITGA7	0.3668	0.0186
OSBP2	0.3677	0.0028
FTH1	0.3707	0.0037
NEK6	0.3713	0.0106
NF1	0.3716	0.0094
SELENBP1	0.3741	0.0053
ANO10	0.3744	0.0028
S100A4	0.3770	0.0185
COL10A1	0.3800	0.0329
AMPH	0.3805	0.0366
TAGLN	0.3807	0.0245
KLC2	0.3831	0.0483
THBS2	0.3849	0.0314
SLC25A22	0.3858	0.0390
LEF1	0.3860	0.0440
EXTL2	0.3895	0.0007
FLAD1	0.3902	0.0364
CD248	0.3915	0.0021
ZNF438	0.3924	0.0025
CD81	0.3928	0.0488
BRD1	0.3943	0.0000
SUGCT	0.3950	0.0194
AP3D1	0.3959	0.0317
P3H2	0.3974	0.0135
SORBS1	0.3976	0.0168
STX5	0.4036	0.0001
RSPRY1	0.4044	0.0069
TMBIM6	0.4046	0.0323
PLPPR4	0.4059	0.0450
AKIP1	0.4109	0.0178
KIAA1671	0.4126	0.0078
PAPSS2	0.4135	0.0127
COA4	0.4138	0.0489
DTNA	0.4149	0.0401
RNF145	0.4166	0.0002

DACT1	0.4176	0.0255
GRSF1	0.4180	0.0469
TSPAN13	0.4198	0.0490
SELENOW	0.4243	0.0190
GALNT5	0.4282	0.0273
ADGRB2	0.4301	0.0361
SAMD4B	0.4345	0.0286
SEL1L3	0.4364	0.0376
FKBP10	0.4379	0.0008
INHBA	0.4402	0.0490
ALPK2	0.4405	0.0017
NPIPA1	0.4422	0.0076
TPM2	0.4443	0.0480
SLC39A11	0.4485	0.0006
ZNF207	0.4486	0.0433
GON4L	0.4499	0.0024
RUNX2	0.4505	0.0411
POU2F2	0.4530	0.0358
TBC1D10B	0.4552	0.0494
ALG13	0.4569	0.0169
TNFSF4	0.4612	0.0169
MGST2	0.4661	0.0366
SULT1A3	0.4661	0.0113
PUM2	0.4668	0.0210
IGDCC4	0.4673	0.0041
MEIS2	0.4676	0.0270
TMSB4X	0.4684	0.0086
DMXL1	0.4686	0.0173
CD4	0.4715	0.0214
PKIG	0.4720	0.0115
IL7R	0.4727	0.0290
CNTROB	0.4735	0.0059
RBM23	0.4750	0.0265
SKP1	0.4774	0.0147
MCTS1	0.4795	0.0070
KLHL5	0.4799	0.0396
FNBP1	0.4842	0.0001
TMEM44	0.4856	0.0344
TPM4	0.4880	0.0086
FBLN5	0.4897	0.0438
AC011448.1	0.4909	0.0282
BOC	0.4923	0.0470
TRIM39	0.4929	0.0041

TAF13	0.4929	0.0416
FAAP24	0.4953	0.0214
SERPINH1	0.4968	0.0209
GLT8D2	0.4974	0.0011
PSME2	0.4975	0.0008
ELMO2	0.4975	0.0060
FAM20B	0.4979	0.0049
PFKP	0.4990	0.0399
AIG1	0.5000	0.0089
SPARC	0.5000	0.0400
NAT10	0.5016	0.0497
HOPX	0.5022	0.0184
CYP26B1	0.5043	0.0486
TRAPPC6B	0.5055	0.0053
CHRDL1	0.5059	0.0178
MAP1A	0.5066	0.0192
OBSL1	0.5071	0.0191
DIABLO	0.5084	0.0238
MYO1B	0.5110	0.0070
LAMP5	0.5113	0.0084
C22orf23	0.5130	0.0466
PTRH1	0.5140	0.0412
ERLEC1	0.5174	0.0102
TUBA4A	0.5177	0.0405
ATXN2	0.5199	0.0305
FBN1	0.5205	0.0219
FAM19A3	0.5207	0.0038
NOG	0.5210	0.0287
DAZAP2	0.5215	0.0484
TINF2	0.5228	0.0000
FAM20A	0.5240	0.0169
SORT1	0.5241	0.0071
DCN	0.5247	0.0410
ZNF624	0.5270	0.0000
CTBP2	0.5270	0.0192
EED	0.5272	0.0069
C9orf3	0.5286	0.0058
MTFR1L	0.5309	0.0028
RAB40C	0.5319	0.0220
SPSB2	0.5320	0.0013
CYHR1	0.5321	0.0025
PEAK1	0.5321	0.0443
ATP6AP2	0.5354	0.0335

PPARD	0.5380	0.0057
MZF1	0.5382	0.0051
CEP112	0.5383	0.0200
EIF4G1	0.5388	0.0375
AC133555.3	0.5412	0.0451
CTNND1	0.5414	0.0338
BHMT2	0.5428	0.0028
C6orf52	0.5430	0.0013
ZNF410	0.5435	0.0128
ACSM5	0.5435	0.0475
FBLIM1	0.5437	0.0401
CALU	0.5454	0.0221
CHPF	0.5468	0.0455
PRRC2A	0.5468	0.0335
ARHGAP26	0.5495	0.0066
RETSAT	0.5499	0.0016
IDH2	0.5502	0.0310
RAD17	0.5505	0.0233
LAMB2	0.5512	0.0478
ATP2C1	0.5512	0.0021
C11orf57	0.5517	0.0403
FAM198B	0.5520	0.0005
DCLK1	0.5536	0.0135
TARS	0.5548	0.0171
GMPPA	0.5566	0.0033
ALOX5AP	0.5571	0.0435
RAB5A	0.5603	0.0003
POLR2H	0.5606	0.0394
P2RX6	0.5613	0.0363
CBWD3	0.5619	0.0139
SSH3	0.5623	0.0255
GPC1	0.5629	0.0411
SSB	0.5633	0.0072
CLSTN3	0.5658	0.0133
MYD88	0.5677	0.0255
GNB3	0.5684	0.0054
DST	0.5685	0.0268
NRG1	0.5717	0.0044
LRRC37B	0.5730	0.0138
NCDN	0.5744	0.0345
BIVM	0.5752	0.0051
NADSYN1	0.5760	0.0281
MKL1	0.5766	0.0023

DZIP1L	0.5775	0.0029
SIK3	0.5782	0.0330
NCBP2	0.5786	0.0291
DDR1	0.5792	0.0082
NKRF	0.5826	0.0265
CTSB	0.5844	0.0230
AHSA1	0.5850	0.0389
ETFRF1	0.5858	0.0139
THY1	0.5868	0.0003
SLC16A7	0.5870	0.0247
STAT6	0.5875	0.0272
UBE3C	0.5876	0.0479
RPL7L1	0.5879	0.0440
TBC1D8B	0.5893	0.0467
VPS51	0.5893	0.0000
IQSEC2	0.5895	0.0258
SLC9A1	0.5906	0.0159
TMEM169	0.5922	0.0334
C11orf63	0.5938	0.0398
MFAP5	0.5939	0.0199
MYL12B	0.5941	0.0256
RARA	0.5946	0.0013
SLC10A3	0.5949	0.0089
GPS2	0.5951	0.0249
DYNLRB2	0.5951	0.0163
HACD1	0.5965	0.0219
JAM2	0.5989	0.0401
APH1A	0.5991	0.0149
NSL1	0.6000	0.0326
TBC1D2	0.6006	0.0374
ZNF226	0.6008	0.0228
RAB34	0.6009	0.0027
EHD2	0.6011	0.0128
HDAC9	0.6018	0.0007
CCIN	0.6018	0.0169
ZNF619	0.6020	0.0119
VASH2	0.6025	0.0267
RSPO3	0.6035	0.0003
RUSC2	0.6045	0.0495
FAM156B	0.6053	0.0143
IL13RA1	0.6056	0.0459
MAN1B1	0.6059	0.0498
SLC39A7	0.6065	0.0052

TLCD1	0.6072	0.0007
FAM213B	0.6077	0.0009
CXorf40A	0.6077	0.0404
COL3A1	0.6077	0.0227
FAM171A1	0.6087	0.0174
VASH1	0.6091	0.0489
GUCY1A3	0.6093	0.0067
FGFR1	0.6095	0.0029
SETD4	0.6099	0.0262
VGLL3	0.6103	0.0016
GANC	0.6105	0.0203
CUX1	0.6109	0.0044
KIAA0391	0.6113	0.0328
GRAMD2B	0.6114	0.0379
MDK	0.6123	0.0030
SPATA18	0.6123	0.0474
TMEM209	0.6126	0.0483
ZNF117	0.6134	0.0060
GTPBP10	0.6146	0.0176
RASSF8	0.6146	0.0052
CCL2	0.6166	0.0143
ACOT8	0.6171	0.0290
PHLDA3	0.6176	0.0371
MIER1	0.6198	0.0168
FHL3	0.6198	0.0385
EFEMP2	0.6200	0.0328
RBM39	0.6226	0.0009
CGB5	0.6236	0.0363
PDGFRB	0.6258	0.0377
CSAD	0.6258	0.0030
SORD	0.6264	0.0304
PCYT1A	0.6268	0.0168
ING4	0.6274	0.0432
ADCY9	0.6279	0.0130
COMMD3	0.6280	0.0007
ING3	0.6282	0.0461
NABP1	0.6285	0.0043
RAB3IL1	0.6287	0.0263
RBM19	0.6291	0.0237
BCAP31	0.6303	0.0176
RPUSD1	0.6312	0.0064
VBP1	0.6319	0.0351
EHD1	0.6327	0.0004

ADPRH	0.6330	0.0002
PTGS1	0.6330	0.0125
PLTP	0.6334	0.0087
CDC25B	0.6339	0.0349
PPP2CB	0.6340	0.0192
NUDT18	0.6347	0.0054
MAP3K11	0.6361	0.0339
C9orf85	0.6363	0.0022
PEX3	0.6364	0.0482
PMS1	0.6368	0.0379
TRAPPC6A	0.6376	0.0005
LOXL4	0.6376	0.0243
BLVRB	0.6389	0.0395
PCMTD1	0.6397	0.0126
NLRC5	0.6425	0.0017
MPHOSPH8	0.6429	0.0057
SERPINA5	0.6430	0.0016
MYL4	0.6433	0.0006
IRF2	0.6434	0.0012
ITGA4	0.6450	0.0298
TMEM189	0.6450	0.0262
BAP1	0.6451	0.0401
NAA40	0.6464	0.0016
SMAD7	0.6474	0.0036
ITGA1	0.6485	0.0164
AMMECR1	0.6487	0.0079
VIPAS39	0.6491	0.0325
TSPAN1	0.6513	0.0488
ACSL1	0.6514	0.0333
NTF4	0.6519	0.0420
MCPH1	0.6521	0.0451
CPT1B	0.6538	0.0098
LRRC23	0.6554	0.0485
PHKB	0.6557	0.0317
PRICKLE2	0.6566	0.0314
DSN1	0.6570	0.0464
C14orf159	0.6577	0.0033
MFSD13A	0.6581	0.0126
PHOSPHO2	0.6583	0.0358
RNF170	0.6607	0.0310
PTGER4	0.6614	0.0335
SHISA5	0.6624	0.0150
ST3GAL4	0.6626	0.0062

APOBEC3F	0.6637	0.0346
TSPAN14	0.6645	0.0140
SRM	0.6652	0.0078
GIGYF2	0.6652	0.0043
GRINA	0.6653	0.0423
ACY3	0.6657	0.0354
USP15	0.6660	0.0309
CTIF	0.6663	0.0361
MRPL55	0.6663	0.0021

Supplementary information Table S2. Glioblastoma related genes were obtained from GeneCard, DrugBank Online and OMIM

TMEM132A	MAF1	ASPM
TP53TG5	MIR130A	ARHGEF7
TMEM14A	IQSEC1	DKK3
TAX1BP3	MIR125B2	CD274
MIR339	MIR125B1	TACC3
RASSF10	NBEAL1	TACC1
FOCAD	NDUFAF2	PXDN
EMC10	CARMIL1	NUDT21
MIR99A	CCP110	MMP24
KLHDC8A	IL31RA	LGI1
NDRG4	SYNJ2	DYRK1B
MIR326	PHLPP1	PROM1
WASHC1	LIN9	RAB3D
TMEM106B	MSH2	PLXNB2
LIN54	BRSK2	BACE1
MIR100	ENAH	OGA
NF1	SEMA6B	ADAM17
MIR181C	GJC2	CDK6
MIR181B2	MIIP	EPAS1
MIR181B1	ING4	MSI1
MIR181A2	MMP25	CDK13
MIR181A1	CHSY1	VTI1B
MIR25	UBAC1	DAXX
MIRLET7C	ANKFY1	HNRNPL
MIRLET7A2	PHF3	NIPSNAP2
SEPT14	APH1B	FABP7
RNF146	APH1A	RBBP9
IQSEC3	PAX6	HIST1H3B
RIOX2	PI15	ADAM9
TTC21B	COQ8A	GLIPR1
VOPP1	CERS1	MAD1L1
MIR128-1	GOPC	ADGRB3
MIR126	KMT2B	ADGRB1
MIR10B	DAAM2	PARK2
MIR9-2	ZNF320	RTKN
MIR21	WDR11	YEATS4
BCL2L12	OLIG2	DMBT1
CPEB4	TRIM8	OSMR
CNTNAP3	PES1	PTEN
LRRC4	KLHL2	VEGFC

HGF	MET	LRRC56
CSF3	HRAS	CHTOP
SHMT2	NRAS	CD44
SLC2A1	PRKCH	ALK
FGFR1	MYC	RHOA
FGFR3	ATM	TNFRSF10B
EGFR	HIF1A	PLAUR
ENO2	GFAP	PDGFA
CTLA4	CASP3	IL13
MLH1	FBXW7	APC
CHKA	EGF	IGFBP2
CTSB	AKT3	KIT
CTNNB1	CDKN1A	TNC
CAPN2	MAPK1	PLAU
BMP4	PRKCA	RPS6KB1
STAT3	PDGFRB	IL13RA2
KLK3	CDK2	LEF1
VEGFA	PRKCI	PDPK1
C1QA	MYCN	MTDH
C1QB	GLI1	SPHK1
C1QC	SRC	CDKN3
FCGR3A	CDKN2B	TGFBR2
FCGR1A	MAPK8	GPNMB
FCGR2A	PTK2	ERBB3
FCGR2B	CDKN1B	EPHA2
FCGR2C	E2F1	FASLG
CDK4	MMP2	C11orf65
HDAC1	IGF1R	MDM4
HDAC2	MAPK3	FGFR2
HDAC3	POLE	VEGFB
HDAC6	NDRG2	NOS2
HDAC8	CREBBP	FLT1
acuC1	CHI3L1	ANXA5
TOP2A	RAC1	RPS20
TOP2B	CXCR4	MMP9
H3-3A	TNFSF10	CHEK2
LZTR1	NES	BMPR1A
CDKN2A	PIK3CG	TNFRSF12A
MDM2	KDR	SLX4
MTOR	BIRC5	FAN1
RB1	DNMT3A	EPCAM
H3C1	PRKCB	MLH3
BRAF	AKT2	PMS1
SEPTIN14	RAF1	SEMA4A

CNTN2	PTGS2	CDK3
ANKH	SOX2	PTPN11
BCL2	THEM4	TUSC3
AGAP2	TNFSF13	SP1
H3-3B	PRKCD	SHH
CCND2	TET1	RAD51
IL2	IL6	CDH1
SYP	BRD2	RFX1
ANGPT2	PLCG1	CBL
GSK3B	GRB2	SLC6A16
TGFB1	GSTP1	ANXA2
AURKB	E2F2	ZEB1
CCND3	CYCS	SERPINE1
IGF1	TSC2	GAB1
STAT5B	ADGRD1	EGFL7
BRCA1	IL4R	ABCC3
IFNG	RHOB	EPHB6
CXCL8	IL1B	TIMP1
STAT5A	FAS	AIFM1
CD34	WRAP53	ZNF263
VAT1	KCNK12	WNT5A
SERPINA3	RND3	BMI1
FOXO3	RHBDD1	ABCG2
VIM	PARP1	BCL2L11
NFKB1	MAP2K1	PIK3CB
MMP7	BCL2L1	MAP2K4
MKI67	IFNB1	NFE2L2
FOXO1	TP73	TSPO
CXCL12	S100B	AQP4
EZH2	SMAD4	TWIST1
GJA1	LGALS1	MAP2K6
IGF2	PIK3CD	ATP23
NF2	PRKCZ	HSP90AA1
FGF2	CDC42	NUBP2
POT1	ERN1	TSPAN4
SSBP2	JUN	BUB1B
AURKA	MMP14	CCDC181
CAV1	FOXM1	CCDC196
NOTCH1	CCNE1	PDGFC
PTN	PINK1	CCL2
CASP8	MTHFR	PTPRZ1
CASP9	ABCC1	SOS1
ABCB1	STAT1	TNF
NKX2-1	EP300	DIABLO

ARAF
PECAM1
RTEL1
PSMD3
SMAD7
SPP1
IFNA1
E2F4
BECN1
IL4
ADM
FAP
DNMT1
PRKDC
HEY1
RHOC
MAPKAPK5
PLOD1
CLEC1A
CARD6
LSM1
ST6GALNAC3
FILIP1
PRR15
PGBD5
ANKRD23
HSPA1A
SAPCD2
YAP1
MAPK14
NBN
CDKN2C
NRP1
IFI27
ALOX5
FPR1
TOP1
FOXG1
IRS1
RPS6KA3
TNFAIP3
FZD6
CFLAR
PDCD4
CA9
FN1
HIF1AN
PIK3R3
TGFA
AXIN1
TRIM33
SPRY2
FZD7
DIRAS3
ILK
ROCK1
E2F3
BUB1
HSPB8
DUSP26
SLC6A17
RPL23
KCNK15
MRPS2
MRPS34
MRPL23
XRRA1
TMSB15A
XRCC6
H3C4
ELK1
CCN1
SIK1
TRIB3
NOTCH2
ITPR3
SOD2
GDF15
KDM4C
TCF3
ERRFI1
TNFRSF6B
ITGB1
MCL1
CSNK2A1
TSC1
MIF
CSPG4
TFPI2
PRMT5
GRP
LRP1
HMGA2
CDK1
RHOD
PIK3R2
ESR1
F2R
PIK3C2G
GPR26
EFNA1
KLF6
ERCC1
FADD
EFEMP1
XRCC3
SNAI2
SIRPA
PKM
XRCC5
E2F5
MPG
HSPA5
ABL1
PCNA
HSPA8
SMARCB1
FZD2
RND2
SMAD2
PROX1
WT1
CHEK1
GDNF
FOXP3
CAPZA2
LIG4
WWTR1
PGF
YBX1
CENPU
TGFBR1

CCN2
ITGAV
DDIT3
POU5F1
RICTOR
CYP1A1
SOX9
TUBB3
RELA
HMGB1
FOXO4
CTSL
NTSR1
IGFBP3
MAP2K3
RAD50
REST
HSPB1
TIMP2
S1PR1
PTPRM
S1PR2
FGF1
NTN1
AXL
STK11
MAPK10
ADAMTS5
CASP7
HOXA11
ID1
HBEGF
CCR5
PRKCG
SOX10
H3C2
EXO1
CCNB1
ERBB4
GLUL
MMP1
BIRC7
JAG1
SOCS3
NDRG1
CSF2
TRRAP
DLL4
TNFRSF10A
TFRC
JAK2
EGR1
PEA15
ADAM10
FREM2
APEX1
SMAD3
HMGA1
ATF5
TLR4
HLA-B
PLK1
NOP53
CEBPB
BAG3
IL10
CCL5
LDLR
MRE11
PRKCQ
IL1A
MSN
ANXA7
PTK2B
ITGA3
KDM1A
EIF2AK3
PTCH1
ABCC4
ETS2
NANOG
TLR9
GADD45A
MAP2K2
PLCG2
MAP2K5
CXCR2
TIMP3
SOCS1
RIPK1
HGFAC
EPHA3
IGFBP4
NQO1
FYN
WWOX
SRRT
HES1
LGALS3
SREBF1
ING1
XIAP
KCNMA1
GRPR
TKT
NT5E
TGM2
ITGA9
ALDH1A3
YWHAZ
KIF11
KLF4
SETD2
CCNA2
ITGA6
HLA-A
TUBG1
PDIA6
CYP27B1
BIRC3
LRIG1
MMP13
ARRB1
RRAS
DUSP19
SLC7A11
H3C3
H3C10
H3C12
H3C7
H3C11
H3C8

H3C6
PGR
CD9
EIF4EBP1
PLEKHG5
PDPN
HDAC9
TNFRSF19
SNAI1
CRYAB
PMAIP1
FGFR4
LRIG2
VHL
CDH2
PPARA
SHC1
HOXA9
EPHB4
CDH5
BAK1
HLA-E
SEC61G
AEBP1
ENPP2
BMP6
ANGPT1
PDCD1
S100A4
EDN1
TACR1
STMN3
PDK2
ITGA5
TCF4
HLA-C
STAT6
SEMA3A
EBF3
ITPR1
CDC20
ASCL1
RPS6
NPRL3
PTPN12
APRT
AGR2
RAN
EDNRB
NGF
RARB
H2AC18
SOD1
ACKR3
STYK1
BCL6
CDH11
IDO1
YWHAB
ALDH1A1
BCL2L2
IGFBP1
ID4
FOS
ADARB1
ATF3
CTSS
FSCN1
LGR5
CD70
ELAVL1
GAPDH
WIF1
NFATC1
ECHS1
ACACB
TUBB
PTPRB
ADAM12
PHLDB1
SLC9A3R1
CLDN5
GCH1
FBXO16
SQSTM1
SLC1A2
ENO1
PAK1
NAMPT
DAPK1
F2RL1
FZD4
PLCB1
PLCB4
S100A8
CREB1
ADAMTS4
SIRT1
BUB3
DRD2
VDR
SOX3
PRKCE
IKBKE
PFKFB3
HSPA4
ALDOA
SLC9A1
CD86
SLC1A3
DHX36
IRAK1
MAPKAPK3
PRKD3
SRPK2
NLK
MED12
DYRK2
BCOR
INSRR
CDC42BPG
GUCY2F
TAF1L
STKLD1
AQP1
NFIA
ASIC1
NCL
PPP1R9B
LYN
ADAMTSL1
NOX4

SLC37A4
CD47
GRIA1
MTAP
DNM1L
PXN
MVP
RASSF1
FMNL1
CLEC5A
CDK7
HOXA10
MTR
NFIB
CXCR1
PA2G4
EPHB2
TRPM8
IL12RB1
RYK
H6PD
NTRK2
USP15
SLC34A2
INPPL1
TRAF2
PTPA
SKP2
STAT2
ZEB2
CSF1
WNT2
IL2RA
TYR
MELK
DVL2
PRRT2
AHR
RALBP1
ABCC5
UHRF1
TNFSF12
S100A9
GSTT1
PTPN1
CD151
TEP1
RECQL4
SOX4
SST
XRCC1
MAOA
TYMP
MAP2
APP
DCT
IL15
PPIB
RB1CC1
PIN1
SOAT1
ACTC1
CYP19A1
RRM2
TRIM28
FSTL1
VEGFD
LGALS3BP
EIF4E
CDKN1C
LPAR1
IGF2BP2
S1PR3
PTPRJ
DOCK1
LDHA
CDC25C
SLC16A1
KLRC3
IL2RB
HNRNPA2B1
BID
BSG
NOS1
PTPRT
FUS
TCN2
GOLPH3
PI3
ACTB
IL24
GLUD1
PTPRD
MCM7
CLIC1
DPP3
GATA3
RMDN3
GNRHR
NRIP1
FZD10
PLCD3
TEK
VIP
PTX3
HK2
LGALS2
SERPINE2
B4GALT3
AHSG
RARA
NR1H2
FTL
MAGED4B
CHRM2
KPNA2
EFNB2
GLS2
QKI
IGF2BP3
NME1
TJP2
EIF3E
PDCD10
H4-16
MRAS
RRAS2
PDGFD
YY1
PTP4A3
GPI
RBPJ

ITGA2
PSMA3
DLX2
USP28
TH
ITGA7
TYMS
SUMO1
CKB
HMOX1
PLAT
CCL20
KLRK1
EPHA5
ARF6
EIF2AK2
ADGRE5
EDNRA
PPP1R13L
NFATC2
LIN28A
OGG1
RNASEL
RNASE1
IGFBP7
DFFB
SH3GL2
G6PC1
S100A6
BNIP3
HSF1
THBS1
KCNH2
PIM1
STK4
MAP3K12
EMP2
NTF3
BACH1
LRIG3
SMO
CREB3
PML
CDK5
TNFRSF1A
NCAM1
NTRK1
CALCRL
CRK
GBP1
USP3
MMP12
LRG1
CAT
HMGCR
ITGB3
EGLN1
CSTB
ADGRB2
IL1RL1
MARCKS
NR2E1
PLK4
TGFBI
PYCARD
PLK5
MAPK11
B4GALNT1
TBX2
HOXA1
PON2
TERF1
DYRK3
S100A2
CDK14
CBX7
SLC35E3
SPRY3
NPM1
MAPK9
PARK7
VCAM1
TRIM24
GSR
PTBP1
SUZ12
GSTM1
ECE1
RUNX1
ACLY
DDIT4
RAMP2
CTSK
MRPL28
PLCB3
PLCD1
FZD5
PIK3R4
PLCB2
PIK3C2A
PIK3C2B
ITPR2
MAP2K7
PIK3R5
SOS2
FZD9
FZD1
MAPK6
FZD3
RHOH
FZD8
PLCD4
PLCE1
CCNE2
PLCZ1
RHOG
ROCK2
TES
PPP2CA
CUL2
PRKN
HJURP
ETS1
LEP
ROBO1
CDK20
LLGL1
IL13RA1
IL1RAPL2
PSEN1
HLA-DRB1
MARK4

NTN4	L1CAM	UBC
PRKAA1	CD14	TNPO1
DFFA	AGO2	TET3
CX3CR1	GAST	SRSF3
CDH13	IL32	CHI3L2
RPS15A	EEF2K	SIAH1
RTN4R	LRP6	DDX6
SETDB1	GATA6	CFL1
NTS	NEFL	ATF4
SHC3	CBX5	SAT1
RPS27A	CHAF1A	IQGAP1
PURA	TNFRSF10C	DDB1
CTSD	G3BP1	RUNX3
GLI2	SLC2A3	CCDC88A
EPHA1	MALT1	CTNNBIP1
TNFRSF1B	TRAF6	SSRP1
MAP3K2	CNOT3	ZBTB18
OLIG1	PTPRA	SEMA3B
TIGAR	TRIO	AMOTL2
VWF	SPHK2	H3-4
TRPM7	VAV3	STOML3
CST3	MGP	LHFPL6
STC1	PHF8	MUTYH
NME4	POSTN	PALB2
DCC	N6AMT1	GLI3
TXN	SLC15A2	SMPD1
AIM2	RSU1	AGER
IL17A	GHRH	HMMR
NTRK3	NEU3	FLCN
IL6R	SLC15A4	LUC7L2
CBX3	SLC15A3	VCAN
CAV2	ALKBH1	FLT3LG
PODXL	CCN3	MBP
SPINT2	DICER1	CALR
FMOD	SLC7A5	PRKAA2
PHLPP2	DUSP6	HDAC7
STC2	YWHAG	WDR5
NGB	STIM1	ACP1
C3orf14	ABCA1	ADD3
CNR2	FOLH1	RBBP4
DKK1	PRKAB1	RBMX
F2	CASP4	NOVA1
APAF1	DPYSL2	NPTX2
CCR3	AGRN	SLC16A4

ST8SIA1	ATP6AP2	IGF2BP1
CPEB1	TRPV1	CD276
HSPB2	ERH	FNDC3B
SEMA3C	PAK2	MACC1
ARHGDIA	TLN1	PRDX1
VDAC1	EIF2A	SIRT2
CHD4	HSPD1	CTTN
EIF4A1	H2AX	HDAC4
RPTOR	ATP1A3	MAPT
LAMC1	ARF1	HPSE
RAI14	ST14	MAOB
CAVIN1	WNT3A	CCL18
CCN4	ADORA3	VCL
SPARC	FDPS	TENT4B
RDX	NPY	TRIP13
CTNNA1	HP	BIRC6
HSPA9	GAS6	AVEN
EZR	SIX3	ODC1
PVR	FRZB	FOSL1
RTN4	SAA1	KITLG
AJAP1	EME1	NOB1
RHOT2	PIBF1	SSTR2
SLC2A4RG	TRIM9	PTPN2
CHTF18	MTA2	ATF1
ZGPAT	RPS11	PFKP
WDR90	LANCL2	CD109
ATP1A1	NOS3	MAP4K4
LIMK1	DDX3X	TK1
RXRA	CNR1	ASIC2
USP7	AREG	CYLD
FGR	RWDD3	EPHA7
CEBPA	IL1RN	PLD2
DCX	RINT1	GNB3
LATS1	ABHD16A	PRLR
SATB1	PPP1CA	ITCH
LGALS8	ADORA2B	USP10
CLDN6	DUSP1	FHIT
SPINT1	PDIA3	FGF5
KLF9	TAPBP	HMGB3
NIPSNAP1	ORAI1	LGMN
DPP4	SRR	TKTL1
KAT2B	ITGB8	ANGPT4
ADAR	HHIP	NAIP
PPME1	TDGF1	COPS6

YTHDF2
ADAM33
DYNC1LI1
MXD1
PLXDC1
RNF112
ANKDD1A
CDR1
DHFR
IL1R1
CCR6
ARG2
CRAT
PAM
LIF
GRM3
PLD1
IKBKB
CASP2
CALM1
FHL2
ANG
IL15RA
FAT1
EMP1
AGTR1
REN
AGTR2
KHDRBS1
CSF1R
MPO
GATA4
CASP6
GLS
WASL
LPO
PMEL
DAO
AR
APOE
BTRC
AQP9
PPP1R12A
NMBR
PSMC4
ADGRG1
STAU1
DAZL
DDR1
NUMB
PPARGC1A
NRP2
INPP5K
F11R
AGK
IL7
OSM
PRRX1
DYNC2H1
NUCB2
SART3
NLRP12
PRNP
ALCAM
CDK9
JAK1
CHUK
SLC12A2
BAD
TLK2
MLXIPL
LTA
EPO
THY1
NRG1
PLOD2
CYB5R3
ALOX12
STIP1
USF2
CSNK1E
WNT2B
MACF1
NEO1
FHOD1
NPRL2
CDC25B
ID3
TNN
TMEM39A
INA
DDB2
XPC
YES1
LIMK2
MCM2
CLDN1
FST
GLO1
CRKL
NONO
UBE2D3
FADS2
RAP1B
PSMB4
CANX
CNP
INHBA
MKNK2
PPP1R1B
UQCRB
ESPL1
TRAF1
CADM1
ARHGEF11
ALDH9A1
IAPP
JUND
TRIM27
APOC1
BAZ1B
ECT2
HDGF
HEPACAM
MBD2
PHF6
SLC8A2
MAML1
HLTF
LGALS9
RFC3
SLAMF6

ERCC6	LAMP1	ATP1B2
POLD1	PLEK	PIAS3
RAD23B	EIF3B	ILF2
FBXO11	IFI30	PDCD6
REEP5	TRIM26	CXCL16
CPT1A	TRIM3	E2F7
VCP	SLITRK1	EMP3
ABCC2	SLITRK3	ILF3
RFC1	SLITRK2	HOPX
FEN1	SLITRK4	SLTM
IGF2R	KNG1	DMD
CRP	HNRNPU	GABRA1
EIF5A	SERPINB2	PGD
SMURF2	HPX	NDST1
DEK	MT-ATP6	CUX1
BBC3	C1QTNF8	FABP4
ADARB2	RXFP1	PREX1
CDC37	MYT1	CD1D
BCCIP	FOLR2	BNIP3L
SLC39A9	G6PD	RIOK2
DHX33	GRN	RIOK1
INF2	PKD2	ADGRE2
ITSN1	WEE1	SLC9A9
RBL1	NCOR1	SYNM
HNRNPC	DAAM1	ADCY7
CDC5L	NR3C1	POLR3B
CEBPZ	KCNN4	PIGQ
BARD1	CAMK2G	HBZ
RAD51C	ITGB4	SRMS
RAD51D	MUC1	DECR2
DDX58	ENPP1	ACOT12
CXCL14	SIRT3	LMF1
SPON1	RPL5	LUC7L
PLA2G4A	ITGB5	SLC16A8
PTPN6	ALOX5AP	RHBDF1
HSP90B1	TP53BP1	DMRTA1
ADCY10	KDM6B	RFX4
TACSTD2	HSPA1L	TCP11L2
PRKACA	NEDD9	GSDMC
FLNA	PABPC1	LIME1
PLG	RAD18	RAVER2
BCAR1	BHLHE40	SNRNP25
SMAD1	KDM2A	HBM
COL6A1	KPNA4	CNEP1R1

WDR24	PLAAT4	COL16A1
HEATR3	PRR13	HECTD1
FAM234A	ADA	TRIM44
PGAP6	SGK1	MYCL
FNDC11	MAX	MAGEA3
ENSG00000264545	MECOM	MX2
KDM5B	PPP2R1A	GK5
PHLDA2	CD46	NUPR1
PAX5	CCR1	MAGEC1
SLIT2	SFPQ	NREP
CYP2B6	GAS1	CREBRF
AXIN2	BRD3	DNMT3B
ARID1A	MAP1LC3B	SLC29A1
COL3A1	BAZ1A	CD55
LAMA5	RNF138	SCNN1A
FBLN7	WNK2	CD59
P4HB	NECTIN2	IRF3
CP	REEP4	NEK9
UBE2I	LMO2	SIRT6
MYO1C	SPRED3	PTGER4
SMARCA5	CA12	WNT1
ATXN2L	MYOD1	DCK
RBM14	ARHGEF26	TDO2
LARP4B	LEPR	EEA1
NUFIP2	CSF3R	ADAM8
GRIA2	GPX1	SELE
PSMB8	PPIA	ST3GAL1
TRPV4	EDN3	DOCK7
ROR2	IL7R	CD163
DAG1	MSX1	ANO1
ENG	PITX1	RPS12
ARNT2	LAMA1	NAB2
USF1	DGKA	HNRNPR
STMN1	NOTCH4	UPF1
THBS4	S1PR5	SLC27A3
BTG1	APLP2	ATP5F1A
HOXA5	IL9	SPARCL1
PCBP2	USP11	VTCN1
TP53I3	SPRY1	ADGRL4
PUM2	CD63	TFPT
IP6K2	DAP3	PRKAR1A
TRIM14	NUAK2	DEPDC5
UCN	PHIP	EPHX1
ZHX1	GPR65	RAD54B

MCC	ELOB	FLT4
MSH4	ATR	GRIA3
STUB1	MERTK	MME
ZC3HAV1	ESR2	CYP1B1
TDRD3	CD36	TP63
ANKHD1	CARM1	EPOR
LARP4	KAT5	ROR1
BCR	IFIH1	CYP1A2
GRIA4	NEDD4	MKNK1
CYP24A1	NR4A1	MECP2
BDNF	LIFR	RAP1A
CD81	TMPO	TRPV6
CHD7	MAP1B	COL4A2
IMPDH2	NR1I3	FOXP2
SIN3A	METAP2	LINGO1
PRKD2	DDX1	NPY2R
CXCR3	ACSS2	CD74
KMT2A	NDUFA13	PARP4
SOX6	TJP1	PCNT
SMURF1	RPS3	MTA1
GAB2	EFNA5	SLC13A5
DHPS	PBK	PRDX4
CPT1C	TRADD	CDC42BPB
KPNB1	RGS4	EFNB3
PARD6A	S100A11	MLC1
RELN	VANGL1	RNF135
TAC1	CCL11	CTHRC1
DNAJA1	CCT3	HLA-DMA
L2HGDH	LARP7	FABP5
PIM3	IL16	GINS1
EIF5A2	STK17A	NMB
IGSF8	PFKFB2	RNF213
HERC3	CD93	API5
IPO7	BLZF1	PCDH10
PARP3	COPB1	PIEZO1
ZFP57	DIP2A	PEG3
PDXP	CCL8	BTG2
HOXC9	S100A3	TUBGCP3
TSPAN32	RPP30	SOX1
DOHH	HOXC10	CCNG2
KIR2DS4	ZCCHC8	PBXIP1
NNAT	S100A5	C7orf31
USP26	ZKSCAN7	HMGN5
SRXN1	ATP2A2	BAALC

USP48
DLX4
IFIT1
MOB1A
PJA2
LALBA
SAP18
CCAR1
AVIL
FRAT1
SAFB2
PSPC1
XAF1
GLYR1
FUT4
MUC13
ST7
RRP8
SRP68
VSIG4
CD24
CMTM3
BATF2
MT-ND4
CMTM1
SLFN5
HES3
MARCHF9
GATA2
TLR1
C1S
MASP2
PKD1
ICAM2
TNFRSF25
SOX17
LY96
TNFRSF18
LACTB
MEN1
CD8A
CYP2C18
CYP4A11
CYP4F3
CYP4A22
TTC4
AP1AR
MYH9
PHGDH
ASS1
KEAP1
DCTN1
STX1A
TTK
CPS1
ATF2
FKBP5
EIF4G1
SERPINH1
SNRPB
TAP2
PPOX
XRCC4
EIF4A3
KRT7
TRAF4
SERPINB5
CNOT2
CDC27
DHX9
CC2D1A
CNOT1
DYNC1I2
GIGYF2
LTBP4
TOP3B
CCL4
GGT7
TNRC6A
CDCP1
FXR2
NKIRAS2
MIB2
AGO1
TNRC6B
ZWINT
ALKBH2
FAM107A
YTHDF1
LSM14A
KANSL2
LSM12
UBAP2
ZNF598
CNOT10
DRAM1
MCRIP1
PRPF19
CFTR
ADK
F10
GSK3A
SCD
MAP3K11
USP9X
CTCF
BRD4
KCNH1
IRF1
PC
SIGMAR1
PTGER3
ABCB6
BMX
EPS8
HSPG2
PPP2R2B
SMC1A
ANGPTL3
CTPS1
CSTA
BLVRA
HINT1
EMX2
GFER
NCOA1
MAK
POU2F1
DISC1
FKBP4
EFNA3
LCN2

GRK5	ACTG1	ADIPOR1
CRABP2	CYP17A1	CTSG
UGCG	ACTG2	MGLL
SORT1	PRSS1	HRH2
TRPM2	EIF2B2	LAMA3
CUL7	MSH3	PRSS8
CLK2	ANXA6	HR
BCAN	PTPRG	HPN
CDH4	AIMP2	MYL9
HMGB2	GALNT12	PLP1
NTSR2	XRCC2	SERPINF2
MAZ	PTPRH	CNDP1
PAPPA	ACER3	BAMBI
MPC1	LRRFIP2	GLUD2
SLC17A7	AFAP1	NMT1
RECQL	RNF43	SLC22A18
SUMO3	ANXA10	USP5
PTPN4	SETD6	RUVBL1
CKAP5	EPM2AIP1	SRGAP1
PSMC2	FCGBP	VANGL2
DPYSL5	ORMDL1	D2HGDH
WTAP	PRSS58	CAMTA1
DNAJB9	HNRNPCL2	CFHR1
CDH19	DNM2	GALNT14
SUMO2	SLC25A4	KDELR2
SUPT6H	ACACA	MNX1
DDX39A	HCK	KIF3A
ARHGAP21	TRPC6	IL18R1
CHST1	B2M	RCC1
RAD51B	PAK4	UBE2S
PYGO2	DVL1	PRMT2
ZFHX4	CUL3	RABGEF1
CILK1	NR4A2	SEL1L
RACK1	SLC22A5	TRPV2
AKIRIN2	TXNRD1	DPM3
ALKBH5	PTS	CDK18
MT-CO2	ADAMTS1	CDKN2D
RCOR2	CR1	EIF4E2
TENM1	SKI	EFNA4
SOHLH1	TCF7L2	EREG
MT-ND1	SCNN1G	IL11
MT-CO3	ACD	RAB25
CCL3L1	CCR2	RNF41
COMT	CD27	SV2B

SULF2	EIF1AD	MVD
ZMIZ1	KCNJ10	PRF1
DOCK4	AVPR2	TCF7
CD99	AHI1	USP2
CCRL2	FLT3	USP13
ATAD2	GRIN2A	TET2
ESM1	GNAS	TFDP1
LRP1B	ADRB2	TLE1
PRMT6	SNAP25	PTPN3
RNF40	CAPN1	PTPN14
GABPB1	FBP1	SPRY4
ING5	DIAPH1	CASP5
HM13	CALCR	IRF2
NMI	GGT1	CTSZ
MCM10	TEAD1	LNX1
PTGFRN	CAMKK2	NR1D2
RAB34	ADORA1	SLC1A5
SESN2	SLC25A13	TRIM25
RNF20	PFN1	TLK1
ZFP36	PROS1	TECR
EIF5B	TAP1	PSMD2
MMP28	ACOX1	CALCA
PMF1	ALDH18A1	BDKRB1
RPL38	IDH3A	CEACAM1
WARS1	PDE1C	B3GAT1
ZFX	MC4R	DLGAP1
ACKR1	SLC7A7	GART
ADGRA2	UCP2	FOXH1
NAA30	UGDH	LASP1
NEU4	TPM2	GHRL
PHYHIPL	PSAP	GJA3
UBAP2L	RPS6KA5	FERMT3
ULBP1	ZYX	HOXB13
EPRS1	VAV1	FNTA
SLC4A1AP	CCT5	LEFTY2
ACP3	CRYAA	PDCD6IP
ATP5F1B	CTBP2	NEUROD2
CHERP	ADIPOQ	NODAL
MRPL49	FLNC	MLF1
SVOPL	GLRX	NFAT5
CCDC9	HTRA1	NDP
ABCC8	PDE5A	PUM1
SUFU	PIP4K2A	SLC5A7
PURG	POMGNT1	TNFRSF14

UBE2T
STAM
PRPF31
NRF1
DGKI
ABCB5
CCT2
CX3CL1
ADCYAP1
DLG3
KRT20
GCLM
HDLBP
HPCAL1
IGFBP6
IL21
LEFTY1
PCBP1
LIMS1
TREH
TRH
ULK4
S100A1
TCF7L1
SETMAR
SRSF1
WNT6
VSNL1
ZBTB20
CACNA2D3
CNOT7
CNOT8
CLSPN
ATG4B
ADI1
CLDN3
DYNC1I1
ECH1
GNL3
GLYAT
IL22
GLE1
CYB5R2
POLR2L
POLD2
PDLIM5
SENP2
PHB2
UTRN
STK26
SMG1
AKT1S1
ATAD3A
FKBP14
IFT57
IBTK
ILKAP
MBNL1
LRRC32
PHF19
SFXN1
RRAD
SYT7
C1QTNF1
CAND1
CD58
BCL2L13
CSDE1
ELL2
EAF2
GOLM1
FRMD6
HBP1
KCNG3
MUC4
NRGN
NPY4R
MTSS1
MAML3
LRRFIP1
MPZL2
LAPTM4B
TNS3
TPD52L2
ST6GALNAC2
RARRES2
SEMA6A
WDR12
SMG7
SMOX
ZDHHC17
ARHGAP18
CHIC2
EIF4ENIF1
FUBP3
FBXO5
GBP2
HAUS6
ORAI2
NEIL3
TCTN1
ZC3H7B
ZFAND3
APLN
BTBD2
CLIP3
B3GAT2
ANKRD17
EGFLAM
E2F8
PAK5
MXD4
MOB4
SNAPC5
ZC3H7A
HELZ
LY6K
MDM1
RC3H1
PGGT1B
PTBP3
SCGB3A1
SOX7
XRN1
YTHDF3
BRMS1L
CEP85
BLOC1S2
ADGRE3
FKBP9
OIP5
SEPTIN9

SKA3
TLE6
RBM11
ST8SIA3
TIPRL
SPAG4
SPDYA
SMG8
SMG9
ZNF131
ARL4C
C11orf49
NUSAP1
MAP7D3
PGAM5
TNFAIP8L3
RNF214
SMAGP
TSPAN17
TMEM168
ATP5F1C
ASPHD2
HILPDA
HAUS5
FBXL14
SEPTIN2
PRRC2C
CNOT9
GML
CNOT11
TUSC1
TTC31
CLEC18B
GATD3
NBPF10
TAFA5
KIR2DS2
SALL1
FOSL2
IDE
GCDH
MLX
C4B
IL18
TARS2
IGBP1
DCLK2
EPS15L1
NT5C
NME6
TCP1
SNRPA
BRWD1
BBS1
GTPBP2
PITPNM1
KLHL20
RBM3
SREK1
IFRD2
RPP38
ALDH16A1
FBXO21
MRTO4
GSDME
PAQR6
F3
ETV6
RNF216
GFPT1
TARDBP
ACVR1
CEBPD
RASL10A
NGFR
C4A
USP4
STRN
OSBPL3
STRN3
KIF15
STRN4
CTTNBP2
CTTNBP2NL
STRIP1
PPM1D
GSN
CBLB
CIC
BAG1
CHN2
PATZ1
XAGE1B
SDHB
PAX3
CORO1A
RIPK3
MYT1L
CD248
RBX1
HAX1
ARHGAP35
ARG1
SLC6A9
OTC
SMAD6
NME2
UBE2L3
P2RX1
TMSB4X
RCC2
RRBP1
CACYBP
AJUBA
CERS6
PMEPA1
CKAP2L
SAMSN1
PLP2
CITED4
ACE2
PLOD3
MST1
DGKZ
TMPRSS2
APBB1
EFEMP2
MB
ARHGAP5
FPGS
MED25
HOXD9

RASSF5	MMP21	HDC
PROP1	ZNF217	IVD
LTB	MEX3A	HTR4
DPYSL4	MMP16	PAK6
LZTS2	DNM1	PCSK7
PGK1	GRIN2B	PDE6G
MMP15	KCNK3	MAP3K9
SFRP1	GRIN2D	MTHFD1
SP100	PBX1	MTM1
ABCD3	MYL2	NDUFS6
SNCA	MSX2	RGS9
SLC7A1	PI4KA	SLC34A1
KLLN	SCN9A	SHMT1
MMP3	PRKAG1	SEC24C
IGFBP5	BAP1	SYT2
TUBB4A	ASNS	APOC3
SELL	MAT1A	KCNH7
NKAIN3	SCN1A	GUCY2D
MYB	PPP2R1B	SCN4B
ARF4	CD3E	SLCO1B3
ACTN4	ATP2A3	PSMA1
RECK	AVPR1A	RAPGEF4
MYBPC3	GDF5	STK10
WRN	LATS2	DBT
ADGRV1	PDE2A	ARHGEF12
ZNRF3	PDE6B	CACNB1
TCHP	MAPK8IP1	ANXA3
DDX5	PDE8B	ERF
RBL2	IL5RA	GPR35
RPL35A	RAD54L	KRT13
EEF2	SFN	FABP1
CLCN3	SLC7A9	FUT3
ERCC2	PON1	LIPH
TFAP2A	ACAD8	IDI1
NCF2	AICDA	KCNQ5
SFRP2	CCNB2	KDM3B
COL1A1	COL7A1	PDE1B
GNAO1	B4GALT1	LTB4R2
PADI4	CYP51A1	PPID
POU3F2	CHRNA5	S1PR4
PPIG	CLCN1	SGK2
PADI2	DUSP3	SLC5A8
PADI3	GALNS	TPSAB1
USP18	GNE	TNFRSF13C

PGAM1
RSPO1
AKAP8
ARHGAP4
AMBP
CDK16
COL5A2
B3GALNT1
GJB5
LIPT1
LMOD1
HBA2
HAS3
PARS2
PAICS
MAPK8IP3
OTOF
SLC22A11
SH3KBP1
SLC7A8
UGT1A6
TSG101
TULP1
PEX10
RNMT
CALML3
ACOX3
AGPAT3
ARHGAP17
ARFGAP3
CCNC
CWC27
CLEC3B
B4GALNT2
BRD1
EHF
EPN1
ETNK1
KCND1
FFAR2
IL17B
GTSE1
ITPK1
NT5C3A
MARCO
MAP3K20
POU4F3
MCTP2
ME3
SLC26A9
SLC4A3
UBE2E2
TRAK1
PROZ
POLE3
SP110
SOX18
TAOK2
CCL28
ADAMTS12
ACOT7
DLX1
GAN
GEM
PCDH8
OLFM1
MRC1
MSC
MGAT4B
KCNS1
HPS5
KIR2DL4
RAB8A
SLC26A1
TRIM22
PSMD1
POLR3F
SLPI
ZNF143
CBX8
DDX3Y
ACY3
ACCS
ARL5B
ABHD11
CAB39
CLNS1A
CLSTN2
ATF6B
AFF1
ITGA10
GLCE
KCTD15
IL17RE
IL22RA2
GJA10
GTF2E1
NYX
MRGPRX2
PLCH1
PAPOLG
NEUROG2
MCOLN3
HIVEP2
KLK14
KRT83
SLC25A25
SLC25A37
RAB26
PNCK
TRAPPC2
USP21
UQCR10
RNF130
SYT3
RASAL1
SNX27
DHX34
AKAP4
BRMS1
BCL2L10
EVL
INHBE
ISL2
HINT2
EXOSC1
HSD17B14
HOXC8
CTSW
IRX3
PACSIN3
NUDT3

LHPP	CLNK	SLCO5A1
LY6H	FBXL2	UBTD1
PPP2R5B	FCRL1	PRRX2
POGK	IKZF5	ZNF773
MYBPH	NUDT11	ZNF792
NACA	HOXC11	TTLL4
NEK10	KRT80	WFDC3
RDH16	SH2D3A	ZNF282
SIRPG	SLC16A5	COX8C
SHPK	UFD1	GPR62
SLC37A1	PPIL4	LARGE1
RBP2	SNAPC3	OR3A1
RHOU	ZBTB22	MORN4
RAX	B3GNT8	MOGAT3
TCF20	ADPRHL1	TRIM34
SNRPC	ASCL4	RNF157
RTN4RL1	BRI3	SUPT7L
ZNF44	KLHL4	RGL3
DHDH	EPHX4	PRPF40B
CARHSP1	HEBP1	SNAPC2
BCL7A	OLAH	ZNF709
BCO2	PPP1R16A	ZFP62
ADPRH	PCDHB1	ZNF197
EMC1	SIGLEC11	ZNF586
GAL3ST1	SLC25A47	ZNF669
FRS3	SLC2A14	ZFAND4
KIR2DL1	ZNF563	C1orf56
GPR45	ZNF16	FRMD1
GADD45B	ZNF366	NAA38
HARS1	ZNF627	UBXN10
KLK12	ZNF230	RABL2B
OSR1	CFAP20	ZZEF1
NCR1	BARHL2	ZNF80
NKX2-6	AGBL4	WDR86
MCTP1	ADGRF4	ZNF721
KRT24	FXYD4	ATP5MC3
RAB21	HELT	FAM89B
PGP	EML2	OR3A2
RHBDD2	CASD1	OR51B6
TFAP2D	MYOZ3	OR7C2
ZNF655	NECAB2	SLC35E4
CALML5	MFSD9	ZNF766
APOO	KRT32	ZNF367
CAND2	NELFE	ATP5MC2

NUP58	RAC2	IRAK3
OR10A2	EHMT2	LAMB1
MSANTD2	PLXNB1	HMOX2
ZNF772	SFRP4	UBE2C
OR51D1	SFRP5	KCNA5
OR56A4	NKD2	HNRNPK
OR13C3	DPYD	PDK1
ABHD16B	FASN	OXTR
H2BC18	DKC1	CCR7
PCDH11Y	ABL2	EGLN2
OCM2	PSMD9	ENPEP
SERPINA2	ELAVL2	SELP
ADGRF2	WDR77	RPA2
PRAG1	SIM2	COX5A
PEDS1	RARS1	CLIP1
VN1R5	COL1A2	FLII
DNAJC25-GNG10	NCF4	HYAL2
CYP2D7	XDH	NUDT1
SERPINC1	EGLN3	LRPAP1
NECTIN1	SOD3	SRI
PRMT1	TCEA3	GPR55
CDCA7L	CASP10	MX1
USP1	GRM5	PDCD1LG2
HELLS	EEF1A2	MRC2
MMP11	CSNK2A2	OXT
STS	CCKBR	ARID3B
THBD	ARHGEF6	ICOSLG
ARNTL	FGF9	PLXNA4
TUBA1B	UTS2	NAP1L4
HOXB7	CAPG	MIA2
INPP5F	FGL2	ZNF207
CXADR	MBD1	MLF2
CD207	BIRC2	TRIM59
CCL26	HNRNPA1	MACROH2A1
PFKM	THRAP3	ELOC
TCF12	HSP90AB1	SLC2A2
CLOCK	NNMT	SMARCA4
PRND	FGF13	CYSLTR2
SCG5	MAGEC2	EHMT1
BMPR2	CTAG1B	SLC2A4
CDC25A	ITGB2	NUMA1
MYBL2	RPS6KA1	SLC2A5
TRAP1	ALB	ADAM19
KPNA1	TF	TRIM21

ELOVL2	UGP2	SENP3
CORO1C	CAPZA1	PRELP
MT3	RPL3	KLK7
BTF3	RPL4	SHPRH
MAGEA1	CAPZB	AIF1
DTL	ICAM5	CTRL
TSHZ3	HNRNPH1	IL33
LCMT1	CAPRIN1	TRIP6
CASP1	HNRNPA3	LEO1
HK1	FAM120A	BCAS3
DCN	PPFIA1	EFS
TNK2	G3BP2	PPP6R1
TYRP1	SAGE1	NSD2
THBS2	PRKG1	VASN
PTGS1	ACTN1	NCOA3
ALOX15	PSEN2	SIAH2
MAN2A1	CSNK1A1	ST3GAL4
KRIT1	ANXA1	ABI3
SOX11	PIK3C3	NEURL1
CAP2	DES	GLI4
CXCR6	MBL2	ZSCAN22
DSE	TNFSF11	ZNF875
EGR3	C1QBP	TLR3
ELAVL4	PPP2R5D	DDR2
NFYA	RAC3	TLR2
ZBTB48	TGFB3	IRAK4
TYK2	SRPK1	MYD88
MAPK7	EIF2S1	SLC6A2
GLB1	KCNA3	TBP
GNAI2	TUBB4B	SERPINA1
MMP10	SDHC	RAB7A
ASAH1	AHCYL1	CSK
DDX41	HYOU1	FANCD2
EWSR1	CXCL10	CCKAR
NDUFS2	DNAJB6	GZMB
SLC25A5	PLXNA1	P2RX7
GIT1	KLK6	TGIF1
NHP2	EPHA6	SET
NEU1	IFNA2	ANGPTL4
SCARB2	MCPH1	CCR4
DAB2	USP22	CDC73
DROSHA	TRPC5	EPHB3
MATR3	CHAF1B	STK38
TRAF5	PPP1R15A	WIPF1

C5AR1	LARP1	DBH
PICALM	RALY	BMPR1B
PTK7	SCFD1	DSP
FABP3	VTI1A	SYK
OLA1	TMOD3	PTH1R
IL12B	DHX15	APOA1
RNF168	ANAPC5	EXT1
SSB	C1RL	CSNK2B
PTTG1	CDK11A	CTBP1
SRP54	BRD8	IKBKG
DCTD	EBAG9	KCNJ1
CCK	LAS1L	NOD2
CYB561	NOM1	MASP1
FHL3	RNF113A	SLC12A1
MRPS22	DEFA4	RPL11
LYVE1	ATG14	RPS19
RANBP9	INTS7	C3
RPS13	IPO4	EXT2
CCNT1	TAF6L	KLKB1
EIF6	NKIRAS1	CYBA
PDLIM1	CSN1S1	IL2RG
RFX3	ACTBL2	SLC11A2
RAE1	BRIX1	SLC25A20
RFX2	NOL9	PTGDR
TIAL1	MRPL4	STAT4
TLR10	MRTFA	C2
CXCL2	PHF5A	DBI
COPS3	PWP1	ABCB4
ELP4	ADA2	C5
IQGAP3	MEPCE	CAST
MLANA	TRMT61A	CYP21A2
POP1	CGB5	CDK5R1
SUPT5H	DCAF1	DLL1
SRP72	CEP162	ERG
ZMYND8	MRPL58	EFNB1
CREB3L2	SEPTIN7	F5
CD200	MTREX	FURIN
CHD9	INTS11	LMAN1
FERMT2	PCLAF	HRH1
IL34	MIB1	HTRA2
GGCT	SDHA	LTA4H
NAPRT	ENC1	PNKP
OXER1	JAK3	SLC27A2
MSL3	NFKB2	PTHLH

SERPING1
SERPINI1
CPE
CFL2
BCL10
CFB
LAMB2
IRF9
FCER2
IL11RA
MINPP1
NR2C2
NOD1
KLK1
TICAM1
PTPRN
PTPRN2
RPL18
SOCS2
ALPI
APOA2
C9
COL6A3
GABARAP
HSPA6
KIF23
MYO1E
NFIX
PER2
ME2
MEFV
CYP4B1
RPS14
TLR6
CD209
C6
DNASE1
ACAA1
ALOXE3
C7
C8B
CCR8
COPA
ALOX12B

ANLN
DLG1
EEF1D
LBP
KAT6A
FBP2
FCER1G
FXR1
GABARAPL2
IL12A
KHSRP
NAA10
PDE1A
OPHN1
POLR2B
P2RX4
KLK2
KLK4
RCAN1
SENP1
TOLLIP
PRL
RBCK1
SAMHD1
RNASEH2A
PSMC5
RPS17
RAB3A
PTPRU
RPL27
TAF15
TAGLN
VDAC2
CCL7
DDX17
CNBP
ADAM23
C8A
CAPNS1
CLK1
CNTNAP1
ALOX15B
ACTR3
ARPC2

BPI
CKS1B
INSL3
IRAK2
FCER1A
ICOS
CXCL1
ICAM3
KDM2B
GFI1
HAS2
PAWR
MLST8
POLR1B
PLXND1
CYTH1
HS6ST1
KLK15
SELPLG
SEPHS1
PABPC4
TNFRSF8
PTGDR2
RFX5
RNF31
SND1
RPS3A
RPL12
RPL26
SERPINB3
RNF8
SPAG9
SRF
ZMYND11
DIO2
DHX30
ARHGAP29
C8G
CD80
CCR9
C4BPA
C4BPB
ARPC1A
ARPC5

BRF1
CLCA1
CLCA2
BCLAF1
FARSA
HS6ST2
HIF3A
FCGRT
LIMA1
LTBR
PDLIM4
METTL3
ULK2
RHPN2
SCGB1A1
STK19
RPL8
RPL27A
RPS16
SERPINB1
ZP1
TSN
DACH1
COPE
DEF6
BOK
CLEC1B
ITGAE
KLF11
HNRNPM
NAT10
PARG
KMT2E
TRIM11
RPL24
RPL30
DEFB1
C1D
DEFA5
ARF3
CD180
BACE2
ADAP1
CLEC12A
GRK3
HRK
KLF8
NUDT6
NUMBL
MUC6
MYL6B
NOP58
NIPAL4
NPLOC4
PDS5A
RBFOX1
NEIL1
SLC25A23
SLC7A4
TRAM1
TNFSF18
TWF1
TWF2
PSD
RGS3
SSBP1
SUPT16H
RPL18A
RPL34
RPS4X
SECTM1
ZNF76
ZNF436
CCL13
CCL1
DEFA3
DEFA6
ADAMDEC1
ACAD11
CCR10
ARPC4
DLG5
IPPK
GPR108
FHOD3
ICAM4
OLFML3
MYO1B
MUC7
MAL
SLC2A11
PLAGL2
SPECC1L
SOCS4
WDHD1
TSLP
ZFP36L2
DDX56
ARPC5L
ANOS1
EFHD2
FRMD4A
IL17C
PAM16
HES7
TNFSF8
TNFSF9
SCGB2A2
TTYH1
DDX28
CCL24
ATG9B
MZF1
DEFB119
FOXJ2
EVA1A
MOB2
LUZP1
SCAF4
RAVER1
RUBCN
TTYH3
ZAR1
H4C1
LY6G6D
STYXL1
TMCO6
FAM98A
DEFB118
MAGEA2
DEFB126
DEFB127

FAM120AOS
H4C14
DUSP4
EPN3
PLPP3
MELTF
RIOX1
ERCC3
UGT1A1
CDC6
CLCN2
AASS
UBE2D1
ABCB7
UBTF
HAVCR2
LTBP1
PFAS
TRMT1
PRKRA
ACTL6A
CDO1
HCLS1
RPL7A
CYTH2
AP1M1
FLAD1
BLVRB
THAP1
CPSF6
CYTH3
CDK5RAP3
SRRM1
ZNF638
NOP2
MAP7
TOMM22
FYTTD1
GPRASP1
GPATCH4
NIFK
TRIM65
TAC4
METTL17
IQCD
H4C15
LOX
MMP19
PAX8
GJB6
TSC22D1
CA2
CTSH
SLC11A1
PDE4A
GREM1
MDK
VTN
FRS2
PFKFB4
ASAP1
MGAT5
RAB3IP
FMNL2
FMNL3
LCK
DRD5
INS
HLA-DRA
GSTM3
HNF1A
TUBA4A
CNTN1
PDE7A
NCK1
WNT7B
PDE3B
AMFR
CENPF
PCYT2
EDN2
SDCBP
TTF2
BLOC1S6
IL20
MAGEA4
SLC38A3
UNC5A
SLITRK6
FOXD1
NECTIN4
SLITRK5
DAOA
UCHL1
CASR
ANPEP
CSNK1D
TPM1
TACR3
ENO3
CASK
IFNAR2
MVK
MARK3
POR
PDXK
TRPC3
ADAMTS13
ADCY3
FGF8
GGCX
NDUFS7
MEF2A
HSD17B4
UNG
PISD
PITX2
RASGRP1
TLR5
ARHGEF1
ACADVL
ACHE
ASL
ATP2C1
AMACR
ADORA2A
CSNK1G2
FKBP1A
GP1BA
ITPA
MAG
PKP2

PHKB
POLG
POLB
POMT1
MYO6
P2RY2
NDUFS1
LAMC2
PRDX5
PRDX6
SLC12A4
TPT1
ST3GAL3
TGM1
SMAD9
XBP1
TRPS1
APTX
AIP
CTSA
COL18A1
ATXN3
AGL
ACSL1
BMP7
EIF2B4
EMD
EIF4A2
GMPS
GP6
FMR1
DPAGT1
NUP62
NUP155
MYO9B
MYO5A
NAT1
NFE2L1
NFATC4
LTB4R
MARK2
PNPLA6
POLR2A
HEXA
LAMA4
TOR1A
UROD
PI4KB
PPP2R2A
RFC2
SORL1
SPTBN1
VAMP1
TAT
CALM3
DAPK3
DCXR
AP1B1
CLPB
ATXN1
ARNT
CHRNA3
GCLC
LMNB2
JAM3
NFATC3
NID1
PLSCR1
SLC4A2
SLC25A19
TRIM32
PLAGL1
PPAT
SUV39H1
TDG
PGM3
SMARCAL1
YARS2
DDAH1
ABI1
ARHGEF3
CDCA7
CLK3
ATG7
AZGP1
ATP13A2
ATXN2
BANF1
ARSG
CLCN5
EDC3
EIF2B5
GABARAPL1
FZR1
KAT7
FBL
GNB2
FOXA1
GTF2E2
ENTPD5
COQ7
CRBN
DOK1
GFPT2
PCM1
NOL3
MANBA
MAPRE2
POMT2
NOP56
MCF2L
SLC25A10
SLC39A4
MADD
SLC4A7
TGFB1I1
TACR2
TSFM
TPP1
UBA5
RIN2
PPM1G
RHOT1
SEC31A
TCOF1
PRPF3
TLE3
SREBF2
VAC14
TMPRSS3
UVRAG
VARS2

XRN2	PDZK1	AMY2B
CPD	SLC35A3	ATP13A1
DARS2	SLC39A7	CHFR
DGCR8	PUS7	BPHL
DNA2	SLC35A2	ASAH2
ADAMTS10	SLC30A9	ELOVL1
ALMS1	RTN2	HOMER3
ABR	USP6	KTN1
CCT7	TREM2	EPB41L2
ATP6V0A1	PLEKHG4	EPS8L2
AGPAT1	PSME1	IFRD1
ADPGK	PSME3	GTPBP3
CTDP1	PROCR	DPP7
FLOT1	PRPF6	DNAJB11
FLOT2	RNF14	PAOX
EXOC7	SARS2	PARP8
ERC1	RABGGTA	NUCB1
EIF5	RPGR	NUP85
EIF4G3	SMN2	NFIC
FTSJ1	SPTBN2	NOP10
GORASP1	WWC1	MICU1
KLC2	SMUG1	MSLN
FOSB	SMARCD3	MAFF
FANCI	TARBP2	PINX1
ICMT	TMEM43	PITPNB
IDH3G	CPNE1	POLD3
DPP9	DBNL	MED15
KLC1	DDX50	MOV10
PAFAH1B3	DHX16	HMGN1
PAPOLA	DPH1	HNRNPF
NKX3-1	ADAM11	KLHL9
MPV17	ABCB9	SEC14L2
MSH5	APEH	PRG2
LTBP3	ANAPC10	RAB6B
LIN28B	AP1G1	SGMS1
MBOAT7	AATF	SH3BP2
PHF1	ABCC10	SHARPIN
PIGB	ACTR1A	RNPEP
PER1	BZW2	TPM4
PASK	CDH10	UPF2
POLI	CTDSP2	UBE2O
POLK	CEP290	UPF3B
KIF13A	CLTCL1	USP19
LAMC3	CEP170	TRIM71

UBE2D4	COMTD1	OBSL1
UNC5C	DCP1A	OSBP2
PKP4	DIDO1	MYO9A
RBPMS	DIP2B	NAPB
SRSF9	DDX59	MRPL42
STARD13	DDX23	NOXA1
PSMC6	ALG13	PGS1
SORBS3	BYSL	PKP3
TAX1BP1	ARL4A	POLDIP3
ZC3H14	CDCA3	PPP4R1
WDR1	CLIP2	PIGP
WDR45	CNN2	LZTFL1
ABCE1	ATG9A	POP4
APBA3	BAG4	NPTX1
CENPA	BAIAP3	KIFC3
ATP8B3	ABTB1	KLHL12
AZU1	BMP2K	PEX12
ELP3	CBFA2T2	TRAPPC10
ETV5	CIZ1	TBCE
FASTKD2	CIRBP	TGS1
GAPVD1	DUSP23	TUBGCP2
LMBR1	ECSIT	TRMT2A
HBS1L	EXOSC9	TSEN34
COPZ1	EDC4	TRA2B
CRIPT	EIF3K	PSRC1
HACL1	ELMOD3	RBM28
MYSM1	FUZ	RNF10
NCBP2	GPRASP2	SSH3
ORC2	IQSEC2	SSR2
MGRN1	KPNA6	SUMF2
NIF3L1	KANK2	TFDP2
MFAP4	GOSR1	STAP2
LAMTOR3	IKZF2	TCF25
RAP1GAP	HIGD1A	TJP3
SAFB	HNRNPAB	THOC2
RIF1	GTPBP4	SURF4
SEMA6D	DPP8	RPL14
STX6	DNASE2	SNX14
SUPT4H1	DOCK9	SLMAP
SFXN2	KRR1	SNRNP70
RPS18	NAP1L1	SF3B2
RALGAPA1	NCBP1	SMTN
SRSF5	NUDT12	ZNF79
TTC3	NUP50	VPS28

TSR1	NEMF	CLSTN3
TMOD1	RAPGEF6	BAG6
TMEM126B	RAPH1	BASP1
ZBTB24	SLC35F2	AHNAK
TSPAN9	SLC39A11	CTDNEP1
DCP1B	SLC41A2	CLDND1
DCP2	SH3GLB2	EPG5
DHX8	TPCN2	FCHO1
AP2A2	UACA	DZIP3
ACAP2	TRIM17	ERGIC3
ACSF2	TUBD1	GNL2
CCDC8	UBAC2	LARS1
BTBD10	UBXN6	KLHDC2
ASB1	PTOV1	IFT80
CXCL3	REXO4	INO80
CENPK	RNF220	GARS1
CENPT	RNPS1	HERC4
CNTROB	SENP5	HNRNPLL
ADAT1	SSR3	FAM3A
CLEC16A	SSBP3	CREBZF
CDK2AP1	TASP1	KLC4
CHCHD3	THADA	JTB
DUSP14	STAU2	OSBPL7
FTSJ3	SNRPG	LIMCH1
EBNA1BP2	SNRPF	MBIP
GOLGA7	SNRPA1	LPCAT1
HP1BP3	SNF8	LRWD1
KREMEN2	SRP14	PHPT1
L3MBTL3	TAF1A	PNMA2
EPB41L5	ZFR	MFSD1
GATC	ZNF281	HIVEP1
IFT74	DCTPP1	SENP7
HSPA12A	DDX27	PURB
GTF3C2	DIMT1	RAD9B
FBXO3	DHX29	SLC38A9
CTDSPL2	DHX57	SIVA1
DNAJC10	ARHGEF28	TNRC6C
ORC3	AP1G2	TEX264
OSR2	ARHGAP11A	TMEM9
NARF	ABLIM3	PHLDA3
POU3F1	BUD13	RBMS3
PPM1M	CCDC28B	RIC8A
MPPE1	ARMC5	THOC3
MTFR1	CEP78	TIMM10

STRBP
SURF6
SNRPB2
ZC3H11A
ZNF346
YIPF1
ZNF12
ZNF189
CPNE8
CPSF7
DALRD3
DCAF7
AGO4
FOXJ3
GLB1L2
GADD45GIP1
DNAJB12
PATL1
OCIAD1
ODF2L
NKRF
LRRC41
PHAX
NOL6
MED26
MPHOSPH9
HIC2
RAET1E
SHKBP1
RRP1B
RUSC1
TBCEL
USE1
TRIM6
U2SURP
TARS1
PIH1D1
PROM2
SSBP4
TBC1D25
TJAP1
RPAIN
RAB43
SEZ6
WDR18
PRICKLE3
RRP12
RSF1
ZNHIT3
TMEM161A
TANGO2
ZNF185
WDR46
CCDC59
ANGEL2
ACTR8
CEP85L
CEP131
CERCAM
AGO3
ARMC10
ARRDC1
CHCHD7
DXO
CDKN2AIP
FBXL6
ENY2
DTX3
FIBCD1
GREB1L
FILIP1L
FAM184A
GCFC2
KCTD2
GSE1
IARS1
DNAJC21
MLLT11
IMP4
NAA60
NPEPL1
NMD3
MIF4GD
NOC3L
MOSPD3
LONRF3
PHACTR2
PNRC1
NOA1
MFSD11
KLHL21
RILPL1
SDR39U1
QPCTL
SIKE1
TRIM46
URGCP
TNKS1BP1
TAS2R14
UFL1
PTCD1
SEC61A2
RNF167
TBRG4
THUMPD3
VPS8
PRDM4
RRS1
ZWILCH
TBC1D10B
TBC1D17
TMCC1
ZBTB11
ZBTB2
ZBTB21
ZBTB44
ZMYM1
ZFP30
ZNF280C
WDR20
ZNF622
YIF1A
ZBTB14
C1orf43
DBF4B
AKAP17A
CCDC66
ANKRD46
ARMCX6
ELP1
CEP57L1
HNRNPUL2

HAUS8	MAP7D1	DNAH14
IFIT5	LTV1	C19orf25
MXD3	MTFR2	C1orf198
NSRP1	NECAB3	C19orf53
MIEF1	MEIS3	CGB3
LSM14B	KLHL29	EMILIN3
MAU2	NEURL4	FAAP24
LSG1	PYCR3	LINS1
PPAN	SLC10A3	FLYWCH2
POP7	TRMT44	ENKD1
P3H4	PRKRIP1	FAM193A
SDAD1	PRRC2A	H4C2
UBFD1	PTPDC1	MVB12A
S100PBP	RPUSD1	LRRC37A3
SCRN3	SNU13	GTF2IRD2B
ST7L	SPIDR	KYAT3
RBM34	TM2D2	NEPRO
RIMKLB	TAMM41	R3HDM4
SNX22	ZNF205	TVP23A
SPATA20	ZCCHC3	R3HCC1
RSBN1	ZNF506	ZSCAN32
RRP9	ZNF568	ZSWIM7
TMEM184A	ZNF573	SRPRA
ZCCHC10	YLPM1	ZNF48
ZFAND2A	ZKSCAN8	CBWD2
TRUB2	ZNF236	CCSAP
ZNF574	C7orf50	CDIN1
WDR91	BUD23	H4C8
C11orf24	CCDC124	FAM210A
EXD2	C1orf35	H4C3
CDKN2AIPNL	ARMCX5	NBPF6
DTWD2	ENDOV	PPP4R3A
EMC4	DNAJC4	SEPTIN8
KIAA1191	POTEE	THAP12
KRI1	LDLRAD4	CIAO3
KBTBD6	SPRYD7	H4C11
FBRS	PRR5L	H4C12
GPR89A	SERF1A	H4C6
EMSY	SUCO	H4C9
CASKIN2	SPOCD1	INTS13
H1-0	ZNF768	POLR2J2
OCEL1	ZSWIM8	QARS1
NOP16	ZNF628	TUT4
MTERF4	DCAF10	PRR14L

JPT1
H4C13
H4C5
OBI1
ZNF841
C8orf82
BMERB1
FBH1
GOLM2
H4C4
RSRP1
WDCP
CBWD5
KHDC4
NTAQ1
SANBR
PABIR2
SAPCD1
TEDC1
DENND10
TEX22
ACP7
GATD1
MCRIP2
TEDC2
ISY1-RAB43
INSYN2A
IQCN
SRGAP2B
ABHD14A-ACY1
NPIPB5
PEDS1-UBE2V1
NUTM2D
TRA
GAGE13
SPDYE16
NBPF26
SMIM31
FTH1
NOTCH3
PRKD1
SMARCA2
CBS
PTGER2
FAAH
MITF
RUNX2
CD40
IRF7
HDAC5
FER
TNFRSF11B
PRPS1
PNP
CTNND1
BCAT1
FPR2
FH
ARHGAP1
DLK1
CRYM
SDHD
TNFRSF11A
DNM3
ATP6V1E1
ABCG1
GAL
COL11A1
APOD
ACSL5
ANP32A
DTNA
NAT2
RNGTT
SDC1
TIE1
WASF2
CEACAM3
MLLT10
MEOX2
SNCG
BCL3
CLDN11
ISG20
GALNT13
NKX2-2
NOX3
MSI2
SKAP2
TIMP4
PRDM2
GALNT9
MT1E
MT1X
MT1A
SLAMF1
RSL1D1
MCTS1
SH3BGRL3
CPQ
SLFN11
DMXL1
CST9
SPANXA1
BTK
RET
MAP3K7
EPHA4
TUBA1A
F7
LMNA
MAPK12
TPH2
IRF5
KRT14
IL6ST
KCNK9
GATA1
MAT2A
PLA2G2A
ABCB11
KCNA1
FTO
ISG15
MATK
RELB
RALA
TUBB2A
TBX3
SMC3
ZIC1
COX6A1

HSD17B10	MTA3	PPIH
FGF14	MEIS2	MRPL12
GPC4	SEPSECS	TNPO3
OAS1	SLC38A1	RBM4
NCOR2	UBR5	VPS33A
LONP1	TANK	TAF2
PPP2R5C	PRPF4	ZMYM2
PIAS1	TAB1	WDR26
PEBP1	TRPC1	ZNF462
MAD2L1	ARID5B	DCBLD2
RALB	AKAP12	ARHGEF16
TNFRSF21	ASPH	CCT8
PRKAR2B	DUSP5	BAG2
SCO2	GJD2	CRMP1
SNTA1	CAP1	GLIS3
CCNK	IFITM3	DPYSL3
PARVA	PCMT1	MLLT1
NUP214	PCSK6	LSM2
MGAT1	NPC2	PHC2
MCM6	MAP4	PELP1
SIX1	MCM3AP	MRPL15
NET1	RANGAP1	HERPUD1
USP24	SLC3A2	RBBP6
SNCB	SH3GL3	PSCA
TIA1	TNR	RBP1
PTPN13	POLR2C	RGS16
SMARCC1	PSMB6	SP2
ZIC2	UTS2R	SMG6
CA5A	TSPYL1	SPEN
CBX2	XPO5	TMEM59
CBX4	ANAPC2	CCL3
CCNA1	ANAPC1	ARID3A
CNTFR	CNTF	CDC23
CHGA	BAG5	BAZ2B
CHD3	CHD8	BMF
COPS5	ELF3	FUBP1
EEF1A1	ELF4	FOXP4
ETF1	GADD45G	GATAD2A
FOXC1	FMN2	IL20RB
KLF10	DNAJA2	MYEF2
NME3	NCAPD2	NOX5
MAP1LC3A	IPO5	MRPS35
MBD3	NUP93	LSM3
PNPLA8	MAGED2	MAEA

PHC3
PLVAP
PBX2
NELL1
MRPS9
SIN3B
TRERF1
USP34
UBR4
RBM15
SCYL2
PFDN1
SMC2
SRSF4
ZMYND10
ZNF644
CORO1B
CCAR2
AKAP11
ANAPC7
ASCC3
CUL9
CHAC1
ANP32B
CHD6
FOXK2
KCMF1
GPN1
ENOX2
CTR9
IER3
MKLN1
NIP7
MRPL1
MCRS1
MDN1
SAP130
RNH1
TUBB8
RMND5A
S100A16
RBM7
SOX21
ZNF687
DIRAS1
EP400
KIAA1549
INTS3
PCSK4
PAF1
OSBPL11
MRPL39
LSM6
MMS19
MFAP1
MED22
SEC16A
RANBP10
TUT1
TMEM18
ZBTB7A
ZNF384
ZNF536
YPEL5
APPL2
IRF2BP1
ETV3
IFNL2
INTS9
MRPS26
SAMD4B
WDR3
ANAPC4
ANP32E
IL31
INTS1
KCTD5
INTS2
MRPL41
MAGI3
MRPL9
MSL1
KNL1
SHCBP1
UTP14A
RMND5B
SUGP2
ZBTB10
ACTL8
ARMC8
GID8
MIER1
LSM8
TRIM45
TEX10
CDC26
BOD1L1
BEX2
EFL1
PCDHGA11
TNRC18
SPANXC
TCEAL7
ZNF609
ZNF787
ZBTB5
ZDBF2
PALD1
NTN3
RELL1
SGO2
TENT2
SPANXD
QSER1
BRINP3
GCN1
INTS14
ITPRID2
SPANXA2
XAGE1A
WASHC2A
GAGE2C
GAGE7
INSR
NEK2
GRM1
HNF4A
CTH
MAP3K1
COL2A1
ACVRL1
ADRB1

FLI1
GNAQ
MST1R
THRA
HPRT1
GRM6
UBE3A
PLA2G6
ATF6
BMP1
ANTXR2
GJB2
ETV1
KDM6A
HPGD
ENTPD1
DLC1
PCCA
NPC1
NEDD4L
SLC19A1
TRAF3
UBA1
TXNRD2
SOX5
PRDX2
NR1I2
TLR7
YWHAQ
ZBTB16
COX4I1
ABCD1
ACVR2A
ALPP
AQP5
AKR1C3
CD79B
CLU
ALDH5A1
BIN1
CISH
CES1
ETFA
LDHB
GMNN
FLNB
FXN
GRM4
IDH3B
GHR
NOG
RAD21
RIPK2
PSMB9
RPA1
PRDM1
VIPR1
AOX1
C3AR1
CUL1
CSF2RA
ATN1
AFP
KLF5
PDE4C
LTF
PPP6C
KDM5C
SLC8A1
TFR2
ULK1
UBE2D2
PTGDS
PROK2
PSMA7
TDP1
TUBA8
CNTNAP2
BCL11A
CES2
HRG
GFRA1
GHRHR
NPC1L1
PPIF
HLA-DPB1
HSF2
SLC16A7
TFPI
TNXB
PRPS2
TFAM
SERPINF1
RNF2
VPS35
DIAPH2
AGRP
BRCC3
CTNND2
CELF2
EIF3F
GRIK4
GRIK5
IL1RAPL1
ITIH4
GAP43
HOXA13
GSTA4
HTT
NUP107
OPCML
MTRR
MBD4
PKMYT1
POU1F1
PLXNA2
PAPSS1
IL3
SLC22A1
SGK3
SKP1
PDGFRL
TG
PLEC
PSMC3
RASGRP3
ST6GAL1
SURF1
PSMD12
RPA3
RPS2
SP3

PRDX3	CSE1L	COX7A2
TSPAN7	BGLAP	DDX39B
DCTN2	CHST15	ARFGEF1
COL14A1	EBF1	CHCHD10
ARFGEF2	GMFB	CLIC3
C9orf72	GNG5	CDR2
DLL3	GDI2	DUSP13
EIF3A	HLA-F	EIF3I
CEACAM5	INSIG1	GNPDA2
EIF4B	PCDH15	ITSN2
GPR37L1	NUBP1	FNBP1
INHBB	MICB	HOXC6
GDF2	NFYB	GTF2H2
FIBP	POLR2J	CXXC5
FGF7	PPP2R5E	FMN1
IFITM1	POLA2	MICAL2
KLK8	MOCS1	NELL2
PBX3	RCOR1	MFF
NPEPPS	RAB18	PDXDC1
MIP	RAB31	RCVRN
POU3F3	SGCB	RAB27B
PMP22	TCERG1	PSPN
NOX1	UCK2	PSMD5
RAP1GDS1	STAB1	RNF139
RING1	RAB38	ST13
SIRT7	SNX1	STX8
TRIM5	SMOC1	STIM2
TUBB6	TMEFF2	PFDN5
PSME2	WBP2	SNX2
PSMB2	ARHGAP24	ZPBP
PSMC1	AGAP1	VPS26A
RBP3	CRHBP	TMEM165
SCAP	BICD1	WDR61
SDHAF2	HES6	WLS
SEMA3F	GPR18	CCL17
SLIT3	OSTF1	CLEC4A
SMOC2	NPAS3	ACBD3
DBN1	MGAT3	CLEC2D
ACTN3	UBA3	CBLC
ARHGAP15	POLR3K	CDH22
AKAP1	RNF144A	CGN
CA3	TLN2	DYNLT1
CXCL11	RRAGA	EIF3G
CEP55	TSPAN8	G6PC2

GMFG
HEATR1
GSPT2
HYDIN
IFITM2
INSM1
NOVA2
LSM5
PIN4
NID2
PREX2
TBL1X
TXNIP
SCGN
SEMA3G
PRPF40A
RPS25
RPL37A
SPA17
SRGAP2
AQP8
CST2
CST9L
ART1
BRF2
BDP1
GPR182
GPS2
GRIN3A
FNBP4
IBSP
PCGF1
PDCD2
POMGNT2
MORF4L2
RANBP6
SAMD9
SGTA
TCFL5
RNASEH2B
STRAP
PFDN2
RPRD1A
SNX6
ZPR1
TMSB10
HMG20B
KAZALD1
ETNPPL
NT5DC2
LRRC15
PPP2R2D
PLEKHA3
MCOLN2
SNRPD3
WBP4
DACT2
DCTN6
DCBLD1
ABT1
ARSH
ASAP3
DUSP11
ELOF1
ERBIN
ERO1A
HTRA4
IFNL1
LHFPL3
OSTC
MMP26
HSDL2
SLC7A13
TC2N
PID1
TSPAN13
ZDHHC18
CSRNP3
GET3
KLK9
NUDCD1
NAV3
SH3YL1
SYF2
ROMO1
TM2D3
ZDHHC23
ZNF554
DHFR2
CACUL1
BCL2L15
CLDN20
MFSD5
WIPF3
TMEM37
TMEM71
TMEM167A
ALPG
ATAD2B
PATJ
BEX1
H2AC4
CIAO2B
TMEM183A
ANKRD36B
ARRDC5
H2AZ2
IRAG1
MAP3K21
TSGA13
PAXX
MACIR
RUBCNL
NANOGP8
SMIM30
PTPRZ2
PHF20
H3F3A
DYRK1A
MSH6
GABPA
ID2
PMS2
BRCA2
BAX
MXI1
FAM46D
PRAF2
STAG2
MAGED4
SPANXB1
PDZD4

MIR222
MIR221
SLC7A3
NLGN3
GPC3
ATRX
GLDC
TP53
TGFB2
KRAS
PDGFB
TERT
KDM5A
ARID4A
BCHE
PDGFRA
PIK3CA
PIK3R1
CCND1
ROS1
ERBB2
AKT1
LRRN2
PPARG
HLA-G
IDH1
IDH2
NFKBIA
MGMT
RPSA
ICAM1

Supplementary information Table S3. The potential therapeutic targets of Chr-A against glioblastoma

CTSD	PLOD2	RBMS3
ITGB1	CLSPN	EHMT2
KPNB1	FLNC	PTTG1
MLH1	AURKA	CAST
PITPNB	DPM3	RBCK1
TP53	ZCCHC10	FDPS
SSBP3	RNF8	PBX3
INTS14	EXOSC1	SMG7
UBAC2	TLK1	PHF19
ACACA	BCL2L1	RNF146
ZYX	FERMT3	MRE11
DIDO1	MIF4GD	FOXP2
PMP22	MRPL23	PHF6
DICER1	MXI1	PTBP1
RNF41	BTBD10	RACK1
VPS26A	ZSWIM7	ZNHIT3
ELP3	CCNC	PRPS2
PARP1	BDKRB1	GMNN
TMEM9	TNFRSF19	CDCA7L
FRMD6	GPATCH4	ATP13A2
PML	HIST1H3B	HBS1L
FZD6	KCNMA1	IDH1
BGLAP	SLC4A2	DDX39A
CDC25C	NUSAP1	MELK
ZMYND8	RPS3A	CREB3L2
RPL35A	EWSR1	CCNB1
ZBTB14	PTK2	CSDE1
PELP1	SCG5	AKAP8
CCT7	CCNA1	IRF3
NIF3L1	KLC4	RNASEH2B
TP53BP1	GOLGA7	SSBP1
PON2	CSNK2B	DCXR
PHC2	CD58	MCTP1
PRKCD	RBMX	CCDC124
CCL24	FANCI	NELFE
SHKBP1	RSBN1	USP21
ZNF236	SLC38A9	PRKAR2B
RND3	PREX1	ANAPC7
WDR61	MYO6	ARMC10
RAD51B	ZNF586	RPL26

SGMS1	SLITRK6	RELB
C3AR1	PIH1D1	PRKCE
ZNF644	SMIM30	DNM1L
RNPS1	SERPINB2	MRPL39
NPC1	RMND5B	ASAH2
RHBDD2	TMEM71	ST3GAL3
CSF3	ZNF185	KLHDC2
CPT1C	DTX3	YEATS4
PLA2G4A	CENPA	PLEC
ZMYND11	FOS	MORN4
ITPK1	ANXA1	PTPN12
INSIG1	RAD51C	LTA4H
PSMB8	NFE2L1	ARHGAP11A
SMN2	PAK4	UBTF
H3F3A	ANAPC10	YBX1
NAMPT	SF3B2	PRPF31
NUMB	ISG20	HELLS
SUPT5H	LSM1	ZNF655
RPRD1A	PFKFB4	MSANTD2
EZH2	HMOX2	GCFC2
FBL	MAPK9	ELAVL2
TAF1A	ECSIT	PAPOLA
UBE2D3	UBE2C	TIMP1
MPV17	FHOD3	HERC4
BACH1	KTN1	ZMYND10
NAB2	SETDB1	USP3
ZBTB44	LDHA	PTK7
TECR	MIEF1	ACTG2
SLC12A2	ANG	SFRP4
SSBP4	RALBP1	BCL2L13
SMURF2	GTF3C2	PRPS1
NEK2	C3orf14	MYL9
RBM4	ING1	CCAR1
IDH3B	PLCD4	CDK4
HNRNPH1	RPS27A	SEPHS1
RPL7A	SPRY2	SUPT6H
MAML3	WTAP	TPM1
NSD2	TSEN34	CENPT
SP2	ERC1	CNN2
TTK	DUSP23	COL1A1
BUB1	EMP3	SLC12A4
HNRNPC	CPNE1	LRRC15
ING5	SERPINE2	MME
CKS1B	WWOX	CLIC3

KLC1
SEC31A
GPNMB
PLXDC1
SNRNP25
C1QTNF1
TNC
PPP4R1
ZFAND2A
LMOD1
HSPG2
PMEPA1
NFATC3
UCP2
URGCP
RHOC
DLG1
ITGA7
OSBP2
FTH1
NF1
S100A4
TAGLN
KLC2
THBS2
LEF1
FLAD1
CD248
CD81
BRD1
DTNA
TSPAN13
ADGRB2
SAMD4B
INHBA
TPM2
SLC39A11
ZNF207
RUNX2
TBC1D10B
ALG13
PUM2
MEIS2
TMSB4X

ATAD2
IL7R
CNTROB
SKP1
MCTS1
FNBP1
TPM4
FAAP24
SERPINH1
PSME2
PFKP
SPARC
NAT10
HOPX
OBSL1
DIABLO
MYO1B
TUBA4A
ATXN2
NOG
SORT1
DCN
CTBP2
ATP6AP2
MZF1
EIF4G1
CTNND1
PRRC2A
IDH2
LAMB2
ATP2C1
ALOX5AP
SSH3
SSB
CLSTN3
MYD88
GNB3
NRG1
NCBP2
DDR1
NKRF
CTSB
THY1
SLC16A7

KDM6A
IQSEC2
SLC9A1
RARA
SLC10A3
GPS2
APH1A
RAB34
HDAC9
IL13RA1
SLC39A7
COL3A1
FGFR1
CUX1
MDK
CCL2
PHLDA3
MIER1
FHL3
EFEMP2
CGB5
PDGFRB
ING4
RPUSD1
ADPRH
PTGS1
CDC25B
MAP3K11
PMS1
BLVRB
IRF2
BAP1
SMAD7
ACSL1
MCPH1
PHKB
PTGER4
ST3GAL4
GIGYF2
ACY3
USP15
DMXL1
STAT6
